# Disentangling the cave centipede *Lithobius stygius* species complex through molecular phylogenetics and redescription of *L. stygius* s. str.

**DOI:** 10.1038/s41598-025-26880-7

**Published:** 2025-11-28

**Authors:** Anja Kos, Nesrine Akkari, Teo Delić, Ana Komerički, Dalibor Stojanović, Maja Zagmajster

**Affiliations:** 1 University of Ljubljana, Biotechnical Faculty, Department of Biology, SubBioLab, Jamnikarjeva 101, 1000 Ljubljana, Slovenia; 2 Third Zoological Department, Natural History Museum Vienna, Burgring 7, 1010 Vienna, Austria; 3 Croatian Biospeleological Society, Rooseveltov Trg 6, 10000 Zagreb, Croatia; 4 Institute of Zoology, University of Belgrade – Faculty of Biology, Studentski Trg 16, 11000 Belgrade, Serbia

**Keywords:** Subterranean fauna, Genetic diversity, Species delimitation, Cryptic species, Chilopoda, Lithobiidae, Phylogenetics, Speciation, Taxonomy, Evolution, Zoology, Ecology, Biodiversity

## Abstract

Distinguishing species is fundamental for obtaining reliable biological insights, yet due to their morphological similarity many species remain undetected until molecular methods are employed. This work uncovers species diversity in the caves of the Dinaric Karst (Europe), shedding light on the understudied cave centipedes. We focus on the taxonomically challenging species *Lithobius stygius* Latzel, 1880, the first scientifically described cave centipede from the Balkans. Multi-locus phylogeny and uni-locus species delimitation based on extensive sampling revealed that many morphologically and ecologically similar populations previously reported as *L. stygius* belong to at least five clades, consisting of numerous molecular operational taxonomic units. We propose some taxonomic changes, including reinstating species *Lithobius luciani* (Folkmanová, 1935) and *Lithobius intermedius* Folkmanová, 1946. By providing a detailed redescription of *L. stygius* based on the type and new material, addressing morphological variability, and providing reliable molecular characterization, we set the baseline for further investigations of taxa concealed under the name *L. stygius*. The species complex represents an interesting case for the study of its evolutionary history, indicating possible multiple cave colonizations. Although disentangling the species complex requires an appropriate molecular and morphological framework, it is invaluable for reliable species identification and recognition of true species diversity.

**Supplementary Information:**

The online version contains supplementary material available at 10.1038/s41598-025-26880-7.

## Introduction

Distinguishing species is a fundamental yet challenging task, crucial for obtaining reliable insights into many aspects of biology, including biodiversity patterns, evolutionary processes, and conservation^1–4^. Despite their importance, a significant portion of global species diversity remains unknown, with many taxa still undescribed due to our inability to distinguish them on a morphological basis, i.e. cryptic species^1,5^. As this issue may arise either from the genuine high morphological similarity between taxa or merely an insufficient approach to studying their morphology^5–7^, some taxa reported as cryptic may be morphologically distinguished with the development of a more rigorous morphological framework^6,8^. At the same time, the advancement and widespread application of molecular techniques for species identification and delimitation^9^, coupled with so-called integrative approaches^10^ have greatly enhanced our ability to recognize hidden lineages and resolve their challenging phylogenetic relationships.

Uncovering cryptic diversity is especially prevalent in taxa originating from less known or hardly accessible habitats^11^, such as the subterranean realm, where incomplete knowledge of species diversity i.e., the Linnean shortfall is still one of the crucial impediments^12,13^. A major part of faunistic diversity in subterranean habitats is attributed to invertebrates, which are generally underrepresented in biodiversity research^14,15^. These communities often host exclusively subterranean taxa with small distribution ranges, which are prone to cryptic diversification^3,16–19^. The question remains, whether this is the case also for taxa with greater mobility, such as cave centipedes, especially in cases, where morphology based research implies large ranges^20^, which have not been tested using molecular approaches.

Centipedes as important invertebrate predators^21^ play an important role in soil, forest litter, and other ecosystems. Out of 3500 extant species^22^, many can be found in caves, but only approximately 60 are exclusively subterranean, i.e., troglobionts^23–28^. The subterranean species share the morphological characteristics such as elongated appendages and reduced pigment and eyes^29–31^. Several exclusively subterranean and endemic species have been described from the Dinaric Karst in the Western Balkans (Europe), which is a global hotspot of subterranean diversity^32,33^. The Dinaric Karst is home to one of the first scientifically described cave centipedes, *Lithobius stygius* Latzel, 1880, and highly troglomorphic representatives, i.e., *Lithobius troglomontanus* (Folkmanová, 1940) and *Geophilus hadesi* Stoev, Akkari, Komerički, Edgecombe & Bonato, 2015. However, after almost one and a half centuries of research, the taxonomy of several centipedes in the region remains insufficiently resolved^29,34,35^. All the issues are also met in the case of the first described and one of the most commonly recorded cave centipedes in the Balkans, *Lithobius stygius,* whose taxonomy and distribution are still largely unclear.

Originally, *L. stygius* was described from Postojnska and Planinska jama, both of which are part of the Postojna-Planina Cave System in central Slovenia^36^. Thereafter, *L. stygius* was reported from across the Balkan Peninsula^26,34,37–40^, contrasting its extremely wide distribution range with the narrow distribution range of most Dinaric subterranean species^3,29,41,42^. The presence of hidden diversity within the species can be expected since *L. stygius* is considered to be morphologically highly variable between populations and has several synonyms^43^. Moreover, recent integrative studies of centipedes have in most cases revealed a greater species diversity than what was previously identified based on morphology alone^17,44^.

Given that the uncertain identity of the populations considered as the *L. stygius* species complex hinders reliable studies on their morphology, ecology, or general biology, we made a first much-necessary step toward revising its distribution patterns, morphology and taxonomic status. First, we used molecular approaches to investigate the relationships between different populations of the species complex and estimate their phylogeography. Second, we studied the morphology of the originally described type specimens as well as recently collected, and genetically evaluated samples from the Postojna-Planina Cave System, to provide a redescription of the species *L. stygius *sensu stricto. Providing a detailed morphological redescription of the type specimens, coupled with reliable DNA reference, sets the baseline for further investigations of taxa currently concealed under the name *Lithobius stygius*.

## Results

### Phylogenetic analyses

**Fig. 1:**
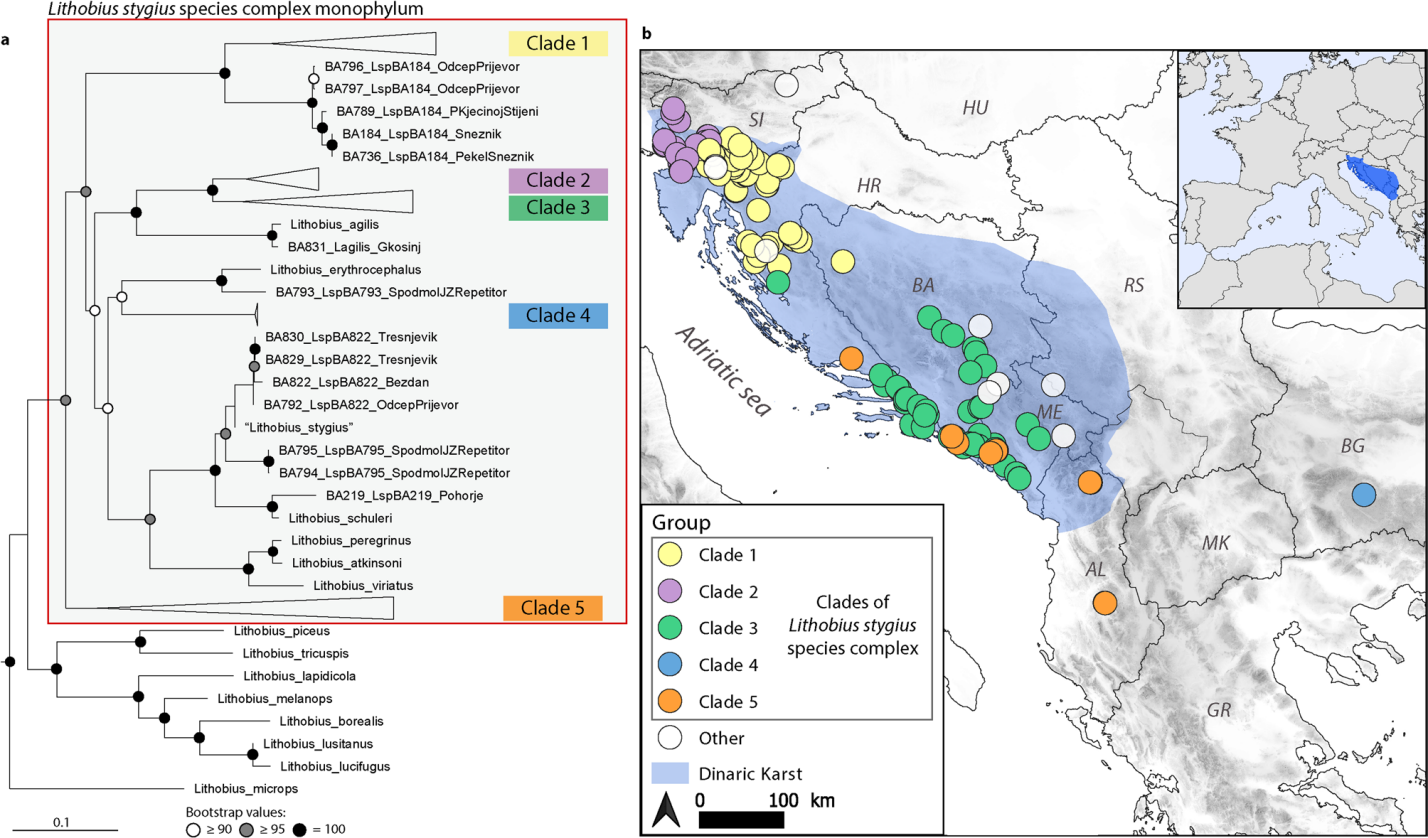
Phylogenetic position of the five recognized clades of *L. stygius* species complex (**a**), and their geographical distribution (**b**). The phylogenetic tree shown was calculated using maximum likelihood approach based on gene fragments COI, 16S rRNA and 28S rRNA (the full tree is available in Supplementary Information online). Labels of the newly-analysed samples of this study are starting with “BA”, with additional information on these samples provided on Figshare^43^. White circles on the map represent newly sequenced specimens that are not part of the five recognized clades of *L. stygius* species complex.

To explore the relationships within the *L. stygius* species complex, we inferred the phylogeny based on cytochrome *c* oxidase I (COI), 16S rRNA and 28S rRNA sequence alignment of 364 specimens, including almost 300 newly collected samples belonging to the target species complex. The analysis revealed that the *L. stygius* species complex encompasses a variety of geographically limited *Lithobius* Leach, 1814 representatives, grouped into five clades. The recognized separate clades (Fig. 1a, Supplementary Figs. S1–S2 online) are mostly well supported by both maximum likelihood and Bayesian inference, receiving high bootstrap and posterior probability values, ≥ 95 and 1, respectively. The only exception to this is Clade 4, as its position is supported by a bootstrap value of 90, but remains unresolved in the Bayesian inference analysis. Apart from the recognized clades, few specimens preliminary attributed to the *L. stygius* complex (BA789, BA793, BA794, and BA795) were shown to be placed in three different positions on the phylogenetic tree and most closely related to surface specimens (Fig. 1a). On the other hand, two specimens collected at the surface (BA956 and BA921) are phylogenetically nested within the Clade 5. All specimens of *L. stygius* complex, together with some surface *Lithobius* species (Fig. 1a), form a closely related and highly supported group “*Lithobius stygius* species complex monophylum”.

Clade 1, which includes the specimens from the type locality of *L. stygius*, is distributed in central and south-eastern Slovenia, north-western and central Croatia, and western Bosnia and Herzegovina (Fig. 1b). The whole clade, completely composed of subterranean populations, forms a sister group to a surface population, herein named LspBA184 (Fig. 1a). Clade 2 is distributed in central and western Slovenia, extending to north-eastern Italy, and partially overlaps with the distribution of Clade 1. Clade 3 is distributed in southern Croatia, central and south-eastern Bosnia and Herzegovina, and Montenegro, with a single, by far northernmost record of Clade 3 representative at the southern part of Mt. Velebit. Clade 2 and Clade 3 form a monophylum, which is most closely related to the surface species *L. agilis* C. L. Koch, 1847. Clade 4 is composed of specimens found in the cave Lepenitsa (Bulgaria) and is possibly most closely related to the surface species *L. erythrocephalus* C. L. Koch, 1847, although this relation is not well supported. Despite a relatively small number of specimens, Clade 5 includes several distantly related populations, sampled throughout a larger part of Southern Dinarides, in an area extending from southern Croatia to central Albania. Its distribution partially overlaps with that of Clade 3 in southern Croatia, Montenegro, and southeastern Bosnia and Herzegovina.

Our study also revealed that the specimens sampled at the surface and identified as *L. stygius* by Ganske et al.^40^ (marked as “*Lithobius_stygius*” in Fig. 1a) only morphologically resemble this species. These specimens are closely related to the surface specimens (population herein named LspBA822).

The “*Lithobius stygius* species complex monophylum” was consistently recovered across all three genetic markers. Based on the mitochondrial markers, namely COI and 16S rRNA, *Lithobius microps* Meinert, 1868 was nested within the monophyletic group, whereas the nuclear 28S rRNA marker placed it in a different position. Overall, single gene trees had lower node support. Position and structure of clades of the *L. stygius* species complex were similar, but some differences in gene-tree topologies were observed. A thorough comparison of single-gene trees is provided in Supplementary Figs. S3–S4 online.

#### Diversity within the *L. stygius* species complex

To identify the extent of genetic differentiation, we performed uni-locus species delimitations of the subset including the *L. stygius* complex monophylum, based on markers COI and 16S rRNA. The delimitation based on COI sequences, applying phylogeny-based method Multi-rate Poisson tree processes (mPTP) resulted in 36 Molecular Operational Taxonomic Units (MOTUs) (Supplementary Fig. S11 online). The distance-based method Assemble Species by Automatic Partitioning (ASAP) on the same genetic marker yielded several results in the top five partitions, suggesting from 97 (best partition) to 52 (fifth best partition) species (Supplementary Figs. S5–S7 online). The subset accounting for potential bias from the overrepresentation of specimens from Planinska jama showed 97 and 52 groups as the best and second-best partitions, respectively. As a result of ASAP, we report 52 MOTUs, as suggesting 97 groups would include several instances of splitting closely related samples from the same location into more MOTUs, which is highly unlikely. The delimitation based on 16S rRNA suggested 31 and 25 MOTUs based on ASAP and mPTP respectively (Supplementary Figs. S8–S10, S12 online).

**Fig. 2:**
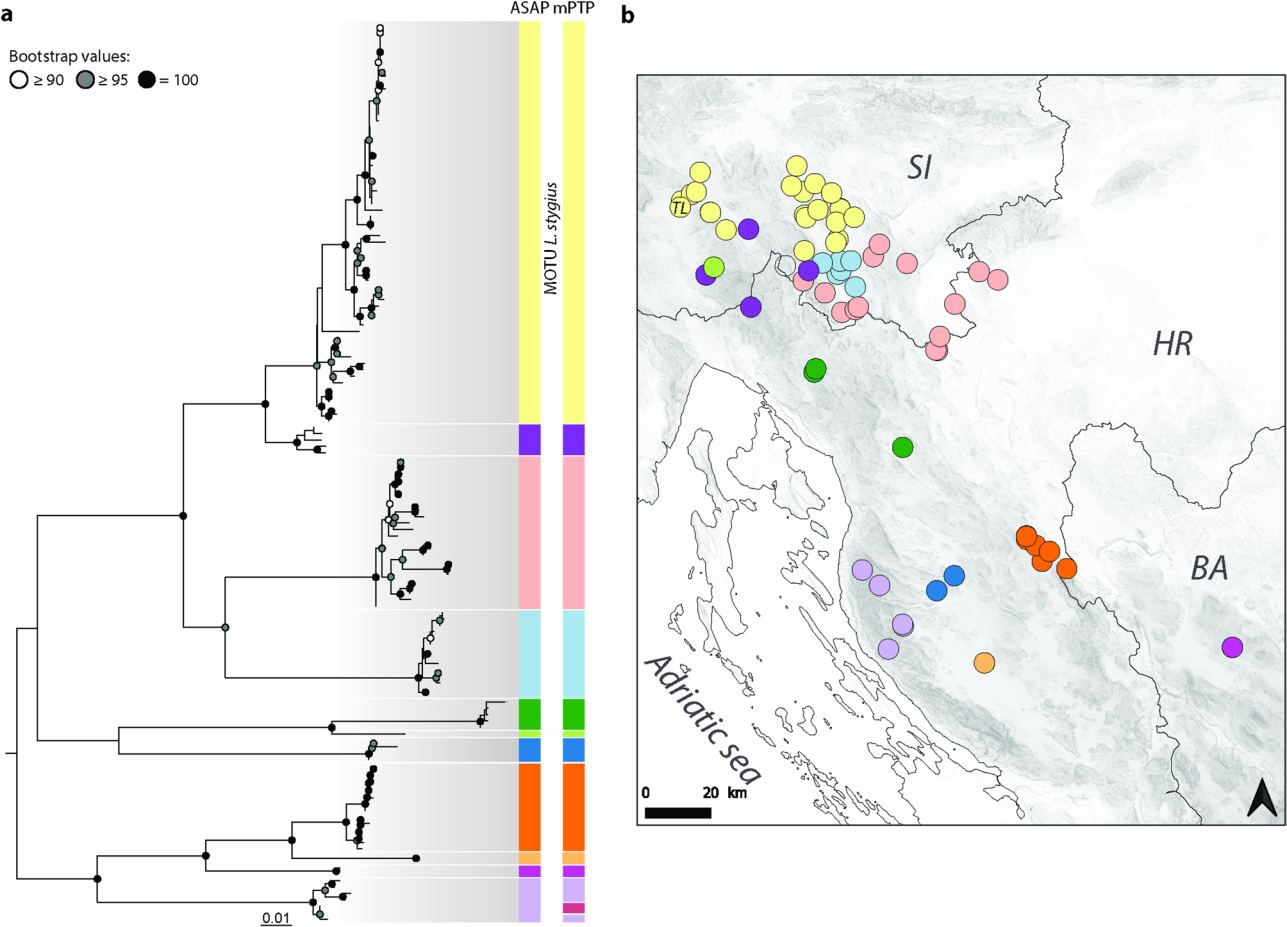
Phylogenetic relationships within Clade 1 of *L. stygius* species complex and its delimitation into MOTUs based on COI sequences (**a**), and their geographical distribution (**b**). The phylogenetic tree was calculated using maximum likelihood approach based on gene fragments COI, 16S rRNA and 28S rRNA. The full tree is available in Supplementary Information online. On the map, colours match ASAP delimitation results and empty circles represent samples without assigned MOTU. TL marks Postojnska jama, which is herein established as only type locality of *L. stygius.*

A closer look into the focal complex reveals that, according to COI-based delimitation, the five clades of the *L. stygius* complex comprised 43 MOTUs using ASAP and 29 MOTUs using mPTP. Specifically, 11–12 MOTUs were detected within Clade 1 (Fig. 2), 1–4 MOTUs within Clade 2 (Supplementary Fig. S14 online), 9–20 MOTUs within Clade 3 (Supplementary Fig. S15 online), 1 MOTU within Clade 4 and 6–7 MOTUs within Clade 5 (Supplementary Fig. S16 online). Delimitation based on 16S rRNA revealed 22 and 20 MOTUs within five clades of the *L. stygius* complex, applying ASAP and mPTP, respectively. Specifically, 9 MOTUs were detected within Clade 1, 2 within Clade 2, 2–3 within Clade 3, 1 within Clade 4, and 6–7 within Clade 5^43^.

#### Taxonomic implications of phylogenetic analyses

The specimens of *Lithobius stygius* from the type locality in Slovenia (Postojnska jama, part of the Postojna-Planina Cave System) fall within Clade 1 (Fig. 2, Supplementary Fig. S13 online), whereas the specimens from the caves, where synonyms of *L. stygius* were described, belong to separate genetic clades (Supplementary Figs. S14–S16 online). Namely, specimens from the caves Vilina pećina (Tuli, Bosnia and Herzegovina) and Poganjača pečina (Grebci, Bosnia and Herzegovina), from where *L. luciani* Folkmanová, 1935 and *L. erythrocephalus cerberi* Verhoeff, 1943 were described, respectively, were positioned in Clade 3. The specimens from Žuljevica (Grebci, Bosnia and Herzegovina) from where *L. stygius intermedius* Folkmanová, 1946 was described were positioned in Clade 5. Samples from Clade 2 are from the area, where *L. illyricus* Latzel, 1880 was described (“osterreichischen Kustenlande”). This species is currently considered as a taxon with uncertain status or a synonym of *L. erythrocephalus*^20,45^*.*

Based on our results we propose the following taxonomic changes:

*Oligobothrus luciani* Folkmanová, 1935 as a valid species *Lithobius luciani* (Folkmanová, 1935) **reinst. stat.**

*Lithobius erythrocephalus cerberi* Verhoeff, 1943 as a synonym of *Lithobius luciani* (Folkmanová, 1935) **syn. nov.**

*Lithobius stygius intermedius* Folkmanová, 1946 as a valid species *Lithobius intermedius* Folkmanová, 1946 **reinst. stat.**

While we re-emphasize that *Lithobius illyricus* Latzel, 1880 is a valid species, we cannot reliably address the status of *Lithobius stygius mazerollensis* Verhoeff, 1937. Based on the distribution data^43^ and comparison of morphological descriptions^46^ this taxon is more possibly closely related to *L. illyricus*.

The *Lithobius stygius* species complex as presented in this work taxonomically corresponds to the species *L. stygius, L. luciani, L. intermedius* and *L. illyricus*, as well as to potentially new species.

### Morphological redescription of the species *L. stygius* (s. str.)

Class Chilopoda Latreille, 1817

Order Lithobiomorpha Pocock, 1895

Family Lithobiidae Newport, 1844

Genus *Lithobius* Leach, 1814

***Lithobius stygius*** **Latzel, 1880**

**Fig. 3:**
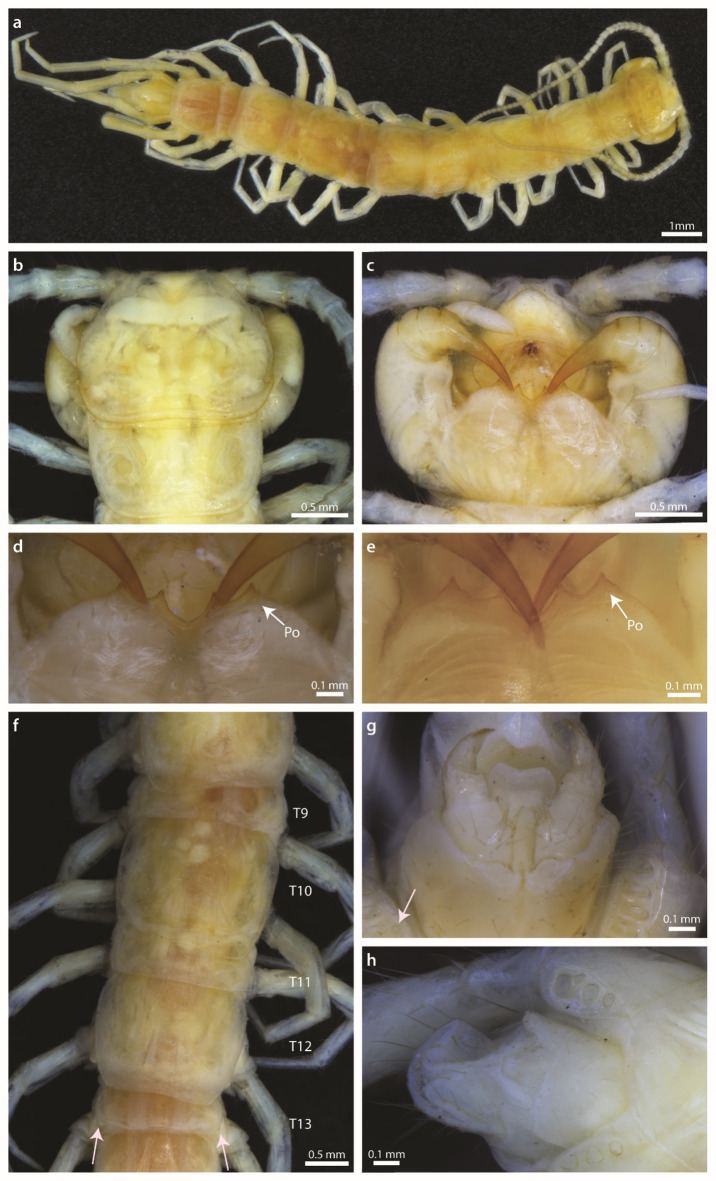
*Lithobius stygius* female lectotype (**a–d, f, g**) and male paralectotype NHMW MY10656 (**e, h**). (**a**) Habitus, dorsal view; (**b**) cephalic plate and T1, dorsal view; (**c**) forcipular segment, ventral view; (**d, e**) close-up of the anterior margin of the forcipular coxosternite, ventral view; (**f**) TT9–13, dorsal view; (**g, h**) posterior segments and gonopods, ventral view. Po, porodont, T, tergite. Arrows point to minute projections (**f**) and to isolated coxal pore (**g**).

**Fig. 4:**
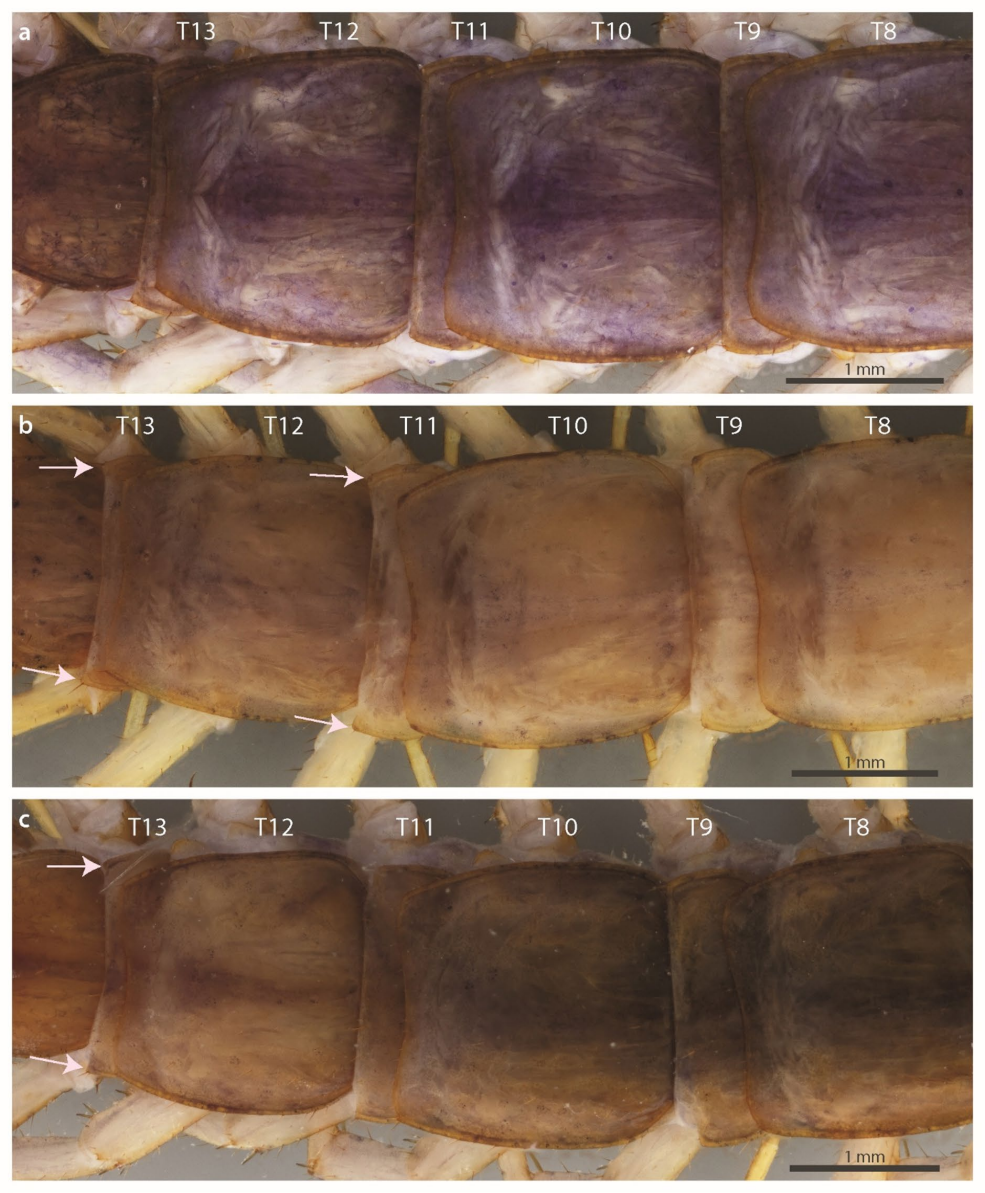
*Lithobius stygius* TT8–13, dorsal view. (**a**) specimen BA578; (**b**) specimen BA842; (**c**) specimen BA965. T, tergite.

**Fig. 5:**
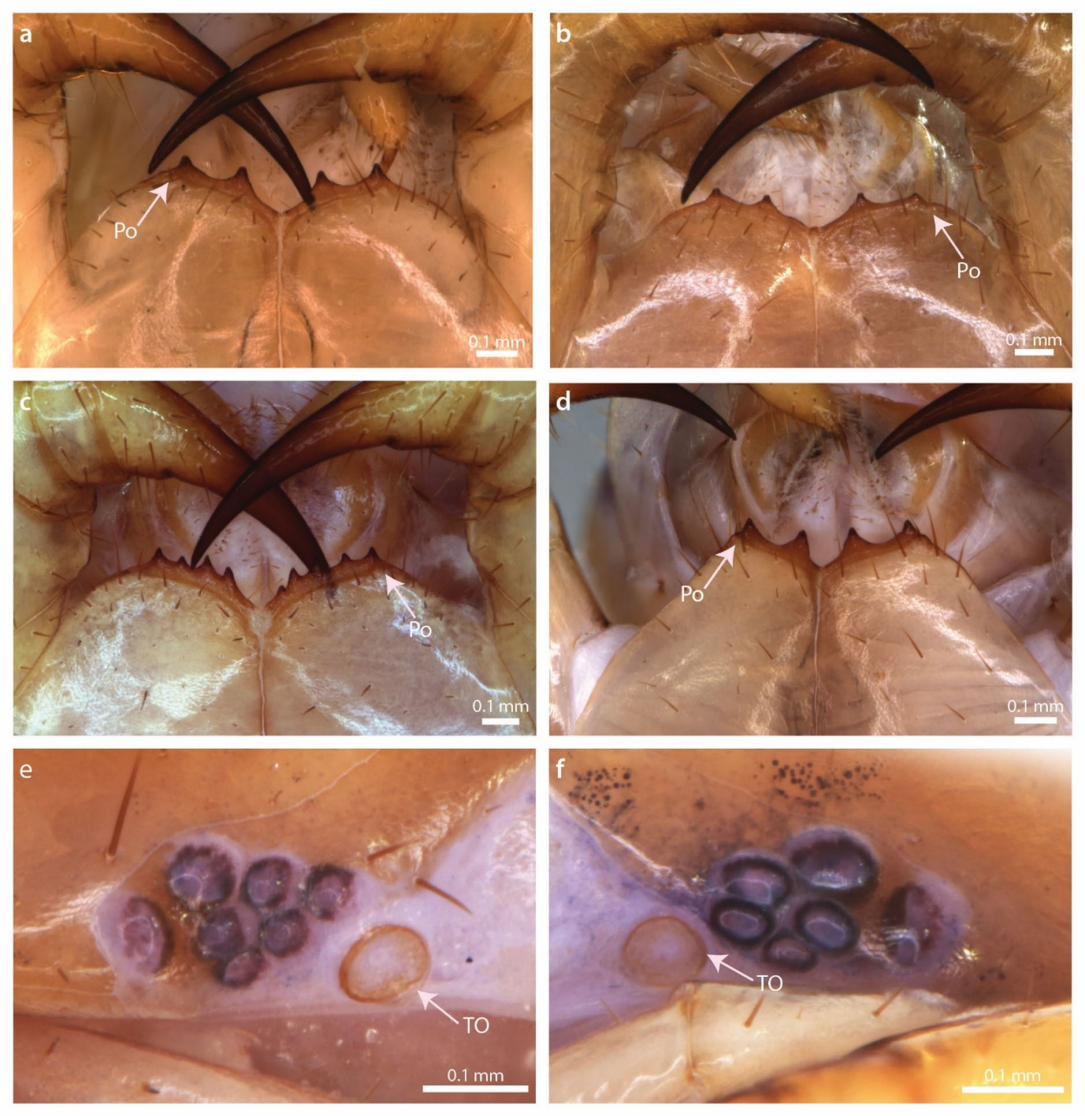
*Lithobius stygius,* (**a**–**d**) forcipular coxosternal teeth, ventral view; (**e, f**) ocellar area and Tömösváry’s organ, lateral view. (**a**) Specimen BA587; (**b**) specimen BA837; (**c**) specimen BA578; (**d**) specimen BA557; (**e**) specimen BA844; (**f**) specimen BA557. Po, porodont, TO, Tömösváry’s organ.

Figures 3, 4, and 5, Supplementary Figs. S17, S18 online.

A list of species citations and information on the material examined is given in the Supplementary Information online.

#### Diagnosis

The species morphologically resembles *Lithobius erythrocephalus* C. L. Koch, 1847 in the following characters: tarsi clearly separated in all walking-legs; without distinct posterior triangular projections on TT9, 11, 13; anterior margin of forcipular coxosternite with 2 + 2 teeth; 15VaC and accessory claw on the ultimate legs present. It is distinguished from other similar species *L. erythrocephalus, L. schuleri* Verhoeff, 1925*, L. illyricus* Latzel, 1880*, L. luciani* (Folkmanová, 1935) and *L. intermedius* Folkmanová, 1946 by having 1 + 5–1 + 6 ocelli, in two or three rows; Tömösváry’s organ larger than seriate ocelli; 33–44 (most commonly 36–37) antennal articles; female gonopod with 2 + 2 elongate spurs and tripartite claw; ultimate legs of adult males without modification; VaC always present on Leg 15 (Supplementary Table S5 online).

#### Description based on the female lectotype from Postojnska jama

- ***Body:*** Fairly robust and evenly broad (Fig. 3a). Body length: 14.7 mm. Mid-body width: 1.8 mm.
- ***Colour:*** After 145 years in ethanol, the specimen is yellowish (coloration likely modified), with paler legs and antennae (Fig. 3a).
- ***Antennae:*** Long (0.48 of the body length) and thin, when stretched reaching posterior part of T6 (Fig. 3a). Composed of 35–36 antennal articles. Ultimate article more than twice as long as penultimate.
- ***Cephalic plate:*** Posterior margin slightly convex (Fig. 3a, b). Cephalic plate slightly narrowing anteriorly, wider than long (ratio length/width is 0.78:1).
- ***Ocelli:*** Depigmented due to preservation, visible only on one side. Round; 1 + 3, 2; posterior ocellus ovoid, the largest.
- ***Tömösváry’s organ: ***Round. Larger than seriate ocelli, but slightly smaller than posterior ocellus. Lying anteroventral to ocelli on the anterolateral margin of cephalic capsule.
- ***Forcipular coxosternite:*** Anterior margin moderately wide, with outer sides higher than inner sides, with 2 + 2 stout triangular teeth of equal size (Fig. 3c, d). Median diastema moderately shallow and wide, V-shaped. Porodont slightly stouter than setae, positioned in front of the lateral edge of outer tooth. Lateral margins of forcipular coxosternite sloping gradually backwards without shoulders.
- ***Tergites:*** T1 with narrower posterolateral side (Fig. 3a, b). T10 widest of all tergites. Posterior border of T8 and T10 feebly concave; posterior corner of tergites T9 round, T11 obtuse, minute triangular projections are present on T13 (Fig. 3f).
- ***Legs:*** Moderately long. Size progressively increasing toward posterior part. Tarsal articulations in all walking-legs distinct. All legs apically with moderately long and curved claws. Ultimate and penultimate legs with accessory claw. Pores of telopodal glands on inner side in penultimate and ultimate legs present. Plectrotaxy as in Table 1.

**Table 1:** Plectrotaxy of *L. stygius*, female lectotype. Spurs present on only one side (either the left or right leg) are given in brackets.

Leg pair	Ventral					Dorsal				
	C	t	P	F	T	C	t	P	F	T
1	–	–	–	–	(m)	–	–	p	a	a
2	–	–	–	a(m)	m	–	–	(m)p	ap	a
3	–	–	–	am	m	–	–	mp	ap	a(p)
4	–	–	p	am	m	–	–	mp	ap	ap
5	–	–	p	am	am	–	–	mp	ap	ap
6	–	–	p	am	am	–	–	mp	ap	ap
7	–	–	p	am	am	–	–	mp	ap	ap
8	–	–	p	am	am	–	–	mp	ap	ap
9	–	–	mp	am	am	–	–	mp	ap	ap
10	–	–	mp	amp	am	–	–	amp	ap	ap
11	–	–	mp	amp	am	–	–	amp	ap	ap
12	(a)	(m)	amp	amp	am	–	–	amp	ap	ap
13	(a)	–	amp	amp	am	–	–	amp	ap	ap
14	a	m	amp	amp	am	(a)	–	amp	(a)p	ap
15	a	m	amp	amp	–	a	–	amp	–	–
- ***Coxal pores: ***4666/4666 separated from one another by less than their own diameter, moderately large. Round (more proximally) to oval or elongated (more distally), on the left 15th leg one smaller pore is located lateral to the main row (Fig. 3g).
- ***Female gonopods:*** 2 + 2 moderately slender acuminate spurs, inner spur slightly smaller than outer (Fig. 3g). Tripartite claw. First genital sternite with seven long ventral setae in two rows. Second genital sternite with up to seven long ventral setae (partly damaged) and four medium long and less stout dorsolateral setae. Third genital sternite with three long ventral setae and two smaller less stout dorsolateral setae. No dorsomedial setae.

#### Male sexual characters of paralectotype NHMW MY10656

First genital sternite with several long ventral setae and concave posterior margin. Gonopod uniarticulated, short, with one long setae (Fig. 3h). Legs 14 and 15 without any modifications.

For a description of the female BA841 from Planinska jama and the male sexual characters of specimen BA965 from Planinska jama, see the Supplementary Information online. Plectrotaxy of the female BA841 is provided in Supplementary Table S6 online.

#### Description of variability

By examining 29 adult specimens, including nine specimens from the type series and 20 newly collected specimens from the Postojna-Planina Cave System, we observed the following variability. The body colour of the newly collected samples ranges from violet-brown to moderately light yellowish-brown, with a medial darker line sometimes present on the tergites (Fig. 4). The head is often lighter, more orangish or yellowish than the rest of the body, sometimes with darker spots on the posterior part or around ocelli. Ocelli are arranged mostly in two (1 + 3, 2) or in a few cases in three rows (1 + 3, 2, 1) (Fig. 5e, f, Table 2). Slight variation was observed in the size of the ocelli and the regularity of the rows. The median diastema of the forcipular coxosternite is generally moderately shallow and wide, V-shaped, but it ranges in some individuals from moderately deep to distinctively shallow (Fig. 5a–d). While 2 + 2 coxosternal teeth were most common, some individuals have combinations 1 + 2, 3 + 2, 5 + 4 (Fig. 5c, Table 2). The posterior borders of TT8, 10, and 12 vary from straight to slightly concave (Fig. 4). The posterior corner of T13 generally has minute projections (never distinct), in some cases they are also visible at T9 and T11 (Fig. 4). The female gonopods have 4–5 dorsolateral setae on the second and 2–3 dorsolateral setae on the third genital sternite. While the majority of females present two gonopod spurs, in one a combination of 1 + 2 spur(s) is recorded. The inner spur is commonly slightly smaller; spurs can also be equal in size. There is a slight variation of spurs elongation. Leg 15 is consistently with VaC. Individual specimens exhibited double spur (8 DpF, 11 DpT, or 13 VaF). A summary of the quantitative characters is given in Table 2, plectrotaxy in Table 3, and all the measurements are available for download from Figshare^43^.

**Table 2:** Quantitative characters of *L. stygius.*

Character	Max	Min	Median	N (measurements)	N (specimens)
Body length (mm)	16.4	10.9	14.3	28	28
Mid-body width (mm)	2.0	1.3	1.8	29	29
Width of T5 (mm)	1.8	1.2	1.5	29	29
Head length (mm)	1.8	1.1	1.5	29	29
Head width (mm)	2.0	1.2	1.6	29	29
Coxosternal teeth	5	1	2	54	27
Antennal length (mm)	9.5	3.8	6.7	46	28
Antennal articles	41	33	37	47	28
Ocelli	7	6	6	41	25
Coxal pores leg 12	5	3	4	55	29
Coxal pores leg 13	6	4	5	55	29
Coxal pores leg 14	7	4	5	56	29
Coxal pores leg 15	7	3	4	56	29
Length 15th leg (mm)	8.2	5.3	6.7	38	23
Length 15th femur (mm)	1.9	1.0	1.4	47	26
Length 15th tibia (mm)	2.4	1.1	1.5	41	23
Female gonopod spurs	2	1	2	25	13

**Table 3:** Plectrotaxy of *L. stygius*. Spur presence/absence was considered stable if present/absent in at least 90% of observations. The unstable spurs are given in brackets.

Leg pair	Ventral					Dorsal				
	C	T	P	F	T	C	t	P	F	T
1	–	–	/	(m)	(m)	–	–	p	a	a
2	–	–	(p)	(a)(m)	m	–	–	(m)p	ap	a
3	–	–	(p)	am	m	–	–	mp	ap	a(p)
4	–	–	(p)	am	m	–	–	mp	ap	ap
5	–	–	p	am	m	–	–	mp	ap	ap
6	–	–	p	am	m	–	–	(a)mp	ap	ap
7	–	–	p	am	(a)m	–	–	(a)mp	ap	ap
8	–	–	p	am(p)	(a)m	–	–	(a)mp	ap	ap
9	–	–	(m)p	am(p)	(a)m	–	–	(a)mp	ap	ap
10	–	–	mp	am(p)	(a)m	–	–	(a)mp	ap	ap
11	–	–	(a)mp	amp	am	–	–	(a)mp	ap	ap
12	–	–	(a)mp	amp	am	–	–	amp	ap	ap
13	–	(m)	amp	amp	am	(a)	–	amp	ap	ap
14	(a)	M	amp	amp	am	(a)	–	amp	p	p
15	a	M	amp	amp	–	a	–	amp	(p)	–

#### Remarks

The original description was based on a type series including 15 adults, six subadults and one anamorph specimen from Postojnska jama and one specimen from Planinska jama^36^. One characteristic noted in the original description and observed in two subadult paralectotypes is the presence of a single female gonopod spur, rather than the two generally found in adult specimens. Herein we designate an adult complete female from Postojnska jama (NHMW MY2085), on which we could verify most taxonomic characters mentioned in the original description of the species, as the lectotype to stabilise its nomenclature. Postojnska jama is here considered as the type locality of the species.

#### Comparison of the examined specimens with the original description

The original description is generally concurrent with the examined specimens, with the following exceptions. Latzel^36^ reports no projections of the tergites (“alle ohne Zahnfortsätze und Eckchen”). However, both on the type and the new material we noted the shapes of T11 and T13 as obtuse and with minute posterior triangular projections respectively (Figs. 3f, 4, Supplementary Fig. S18 online). There was also a difference in the number of coxal pores, as the original description reported a maximum of five coxal pores, but the designated lectotype has six pores, of which one is considerably smaller. In addition, on one of the 12th legs of specimen NHMW MY4049 we observed five rather than reported maximum of four pores. With the examination of the 20 new specimens, we observed variation greater than that reported by Latzel^36^ in: (i) the number of coxosternal teeth (in addition to the reported combination 2 + 2, which is most common, we observed combinations 1 + 2, 3 + 2, 5 + 4 in few specimens, with no molecular difference between the latter specimen with unevenly developed teeth and specimens with 2 + 2 teeth); (ii) the number of antennal articles (reported number is 35–41, we observed a minimum of 33 articles); and (iii) the number of coxal pores, where we observed higher maximum values (for the 12th leg five rather than four, for the 14th leg seven rather than six, for the 15th leg seven rather than five) and the lower minimum value (for the 14th leg four rather than five). We did not observe additional variability in the number of ocelli or differences in plectrotaxy (Latzel provided information on the 1st, 14th and 15th legs). We did not observe any specimen (including types) with only five ocelli as reported in Latzel^36^ arranged in 1 + 2, 2. However, the type specimen’s ocelli are indistinguishable after the long stay in ethanol.

#### Distribution and ecology

The species *L. stygius,* herein considered a MOTU that includes its type locality Postojnska jama, is distributed in the southern part of central Slovenia. It is a troglobiont, known from caves only. There are no taxonomically reliable findings of this species in surface habitats, although the area has been intensively sampled^47–50^. The species is mostly found near the entrances of caves, where there is commonly a lot of organic matter (e.g., decayed wood, leaf litter, faeces).

## Discussion

Investigating the *L. stygius* species complex highlights the challenges of uncovering hidden species diversity in morphologically similar species, where the use of molecular methods is an established practice^1,3^. This approach is particularly crucial in subterranean environments, where cryptic speciation is especially common^5^. Previously, *L. stygius* was considered a morphologically variable species with an unusually wide distribution for a subterranean species. However, molecular analyses based on extensive sampling revealed that the populations reported under the name *L. stygius*^43^ do not belong to a single species, but rather to a species complex. Moreover, the complex is comprised of taxonomic units that can be assigned to several molecular clades (Fig. 1), emphasizing the complexity of lithobiomorph centipedes’ morphology, phylogeny and ecology. This is further demonstrated by few samples (BA789, BA793, BA794, and BA795) that were not part of the five recognized clades of *L. stygius* species complex. Additional sampling is needed to clarify whether they represent additional clades of the complex, or surface *Lithobius* species. Finally, our findings underscore the importance of molecular tools for accurate species identification, as both closely and distantly related clades share similar morphology and ecology.

The *L. stygius* species complex represents an interesting object for further studies on the evolutionary history of the group, suggesting multiple, independent cave colonization events. This is supported by the presence of at least five phylogenetic lineages with preferences for living in subterranean habitats, with the surface species nested within. Such phylogenetic structure is suitable for further studies that could, when supplemented with morphological characters, address the potentially convergence present in this group or describe troglomorphic characteristics in a phylogenetically explicit framework. On the other hand, the cave environment might have different significance for different taxa within the recognized clades of the complex. Various levels of cave dependence are indicated by: i) slight differentiation of morphological characters, which are considered to be related to the degree of adaptation to subterranean habitats, e.g., different number of ocelli and size of the Tömösváry organ^34,37–39,46,51^, and ii) fieldwork observations^43^, where specimens of some populations were collected almost exclusively in the deeper part, whereas others were found in the entrance parts, or, in a few exceptions, also outside. Caves present an important habitat also for several surface or non-obligate cave (troglophile) centipedes^26,34,52^. Furthermore, caves were shown to act as refugia for some taxa during some periods of the year^53^. On the other hand, in line with a climate-relict hypothesis^54^, periods of unfavourable climatic conditions outside caves might be an important driver of cave colonisation. Within clade diversification might be connected to allopatric speciation within the karst massif, which likely corresponds to the Pleistocene glaciations^19,55^, or events in the Miocene Epoch, e.g., the Messinian crisis, orogenic activities in the region and the existence of the Dinaric Lake System^55,56^.

The phylogeographic patterns of the *L. stygius* species complex are in line with those observed in other subterranean taxa of the Dinaric Karst. The differences in the distributions of some clades between the northern and southern parts of the Dinaric Karst are similar to the different distributions of cave amphipod and beetle species^42,57^. The observed spatial overlaps between some pairs of clades were similarly reported in cave amphipods^3^. Even within-clade spatial genetic breaks (e.g., in Clade 1) were similar to those reported for subterranean spiders, pseudoscorpions, beetles, and millipedes in the area^18,58–60^*.* Additionally, the high genetic diversity and its patterns within Clade 3 are comparable to those observed in cave springtails and beetles^19,42^, and the clade is situated in the area with the highest subterranean diversity^41,61^. On the other hand, some clades also extend over the borders of the Dinaric Karst. All these comparisons and overlaps in patterns indicate a common evolutionary history of the different subterranean taxa in the Dinarides, especially in the terrestrial, but also in the aquatic domain.

The inference of the *L. stygius* species complex phylogeny together with species delimitation algorithms revealed greater diversity and endemicity than previously known. This further supports the Dinaric Karst as a subterranean hotspot^32,33^ and the Balkan Peninsula as a region harbouring a great diversity of centipede species^62^. The two delimitation algorithms used, both ASAP and mPTP, were mostly concurrent both for COI and 16S rRNA based analysis, with a few exceptions. Especially in COI based delimitation, in a few cases the distance-based ASAP method splits MOTUs suggested by the tree-based mPTP. This occurred even though we considered the ASAP score delimitation suggesting a threshold of 3.9%, which was more conservative than the highest-ranked delimitation, suggesting a threshold of only 0.9% (Supplementary Fig. S5 online). In comparison, Bharti and colleagues^63^ reported intraspecific distance in centipedes to be in the range of 0–17.1%, with an average of 7.2%. In relation to COI, delimitation based on 16S rRNA suggested a lower number of MOTUs, especially within Clade 3, where only 2–3 MOTUs were suggested in comparison to COI based delimitation that suggested 9–20 MOTUs. In general, the differences in results depending on the algorithm used are common, as each method has some biases and assumptions, and their efficiency is influenced also by the specifics of the dataset and biology of species^64–67^. Thus, the use and comparison of multiple algorithms are recommended^65^. Generally, distance-based methods (e.g., ASAP) tend to be more conservative relative to tree-based methods (e.g., PTP)^65,67^. Among the tree-based approaches, the mPTP has been shown to be both more conservative and more accurate than the PTP^67^. The scoring of COI-based delimitations suggested by ASAP (Supplementary Fig. S5 online) in our case highlights the need for a critical choice of most relevant partition based on existing biological knowledge, rather than the naïve use of the ASAP-score^66^.

*Lithobius stygius* is a textbook example of a taxonomically challenging species complex, for which our results highlight the past struggles to correctly taxonomically assign populations*.* Based on the molecular information, we clearly show that out of many populations reported as *L. stygius*, only the narrowly distributed MOTU named “*L. stygius*” includes the specimens from the type locality of the species *L. stygius*. Furthermore, two previously synonymized names could be removed from synonymy and the corresponding species could be made valid again, and several potentially new species were identified, for example, populations reported from the Bulgarian caves^34^. Different taxonomic discrepancies surrounding *L. stygius* are most likely a consequence of the high morphological interspecific similarity between the taxa hitherto assigned to it, coupled with their intraspecific variability. This becomes further complicated when considering the morphological characters traditionally used for the taxonomy of the group, some of which are clearly affected by ontogeny. They are further compounded by other shortcomings, such as poor species descriptions, unclear definitions of type localities, and presumably lost or destroyed type material.

To overcome these shortcomings and provide the basis for an unambiguous taxonomy of the species, we provided high-quality morphological and molecular descriptions of the species *L. stygius* (s. str.). This contributes to the existing lithobiomorph-centipedes taxonomy, which still heavily relies on morphology^24,68^. It is also useful for species determinations for the researchers without access to genetic laboratories and facilitates taxonomic knowledge across the research community^69^. By updating the morphological description of *L. stygius* based, not only on the type material, but also on recently collected specimens from Postojna-Planina Cave System, we significantly complemented the original species description^36^, and the study of species variability^70^. With the molecular data and the information on the morphological variability of *L. stygius* in hand, we could reinterpret the literature descriptions of taxa previously considered as its synonyms. The reported morphological differences between taxa were previously attributed merely to intraspecific variation, but we believe that they indicate the morphological distinctiveness of the species^34,37–39,46^. However, their scope of variability across all populations, and their taxonomic significance remain to be tested. For example, the presence of 15 VaC spur is generally considered stable and taxonomically very informative^46^ but was considered variable by Stoev^34^. This spur is present in *L. stygius, L. illyricus* and *L. intermedius*, but not in *L. luciani*. On the other hand, characters such as the shape of structures, e.g., coxosternal teeth, gonopods or their number e.g., of ocelli and antennal articles are useful for taxonomy despite their variability^46,71^. This is also the case for the arrangement of spurs on the legs, where we observed a surprisingly pronounced difference when comparing our findings to those reported by Matic and Stentzer^70^, both referring to the *L. stygius* specimens from Postojna-Planina Cave System. Specifically, they noted the presence of the VaF spur starting from the 11th leg, whereas in our specimens it is present mostly as early as the 2nd leg. The suitability of selected morphological characters for species delimitation should be adequately addressed with a morphological framework accounting for interspecific variability^17,44^, or new phylogenetically informative characters should be identified^72,73^. Morphological characterisation of the species considered part of the *L. stygius* species complex should be coupled with their taxonomic revision and follow a procedure as for *L. stygius* s. str.. Additional steps needed include examining type material from natural history collections or collecting fully preserved adult specimens for the designation of neotypes in cases of *L. luciani* and *L. intermedius*, where the type material is lost.

In morphologically highly similar taxa such as the *L. stygius* species complex, molecular tools have proven to be particularly valuable for setting the first important steps in resolving taxonomy and highlighting the hidden scope of species diversity. Patterns of small-scale genetic differentiation and cryptic speciation observed in this group and common in subterranean invertebrates^3,18,19^ offer a novel opportunity for exploring the processes driving species diversification. This should be in future accompanied with integration of morphological data, which holds potential for better understanding of morphological differentiation in relation to speciation or colonization of cave environments. On the other hand, combined with the inaccessibility of their habitats, such taxa present a challenge for taxonomic research and conservation efforts^13^. Despite these difficulties, they represent an important and vulnerable part of global natural heritage and should therefore not be overlooked^74^.

## Materials and methods

### Study area and datasets

This study focuses on representatives of the *L. stygius* species complex, for which we have considered morphologically similar specimens reported from caves throughout the Balkans^43^. We analysed specimens morphologically similar to *L. stygius* and all its synonyms (Supplementary Tables S4–S5 online). Newly gathered individuals were assigned to the complex based on the morphological characters: specimens of genus *Lithobius* with tarsi clearly separated in all walking-legs; without distinct posterior triangular projections on TT9, 11, 13; anterior margin of forcipular coxosternite with 2 + 2 teeth, and an accessory claw present on the ultimate legs. Some characters, like the presence of 15VaC spine, number of ocelli or number of antennal articles, were reported to be variable in some populations^34^, so we included the additional criterion of ecology, considering only individuals collected in caves.

We included all available samples from the known geographic range of *L. stygius*, including specimens from the caves, where some *L. stygius* synonyms were described. Furthermore, inclusion of additional literature and new genetic information of other representatives of genus *Lithobius* enabled us to position the *L. stygius* species complex within the wider *Lithobius* phylogenetic framework.

We used the assembled material for (i) phylogenetic analyses of the broader *L. stygius* complex, hereafter referred to as the molecular dataset, and (ii) morphological analyses of the species *L. stygi*us (s. str.), hereafter referred to as the morphological dataset.

The molecular dataset includes sequences of altogether 364 specimens, including 305 newly collected samples from 153 localities, mostly caves^43^. Among them, 295 belong to the *L. stygius* complex, while 10 are surface *Lithobius* species presumably related to the *L. stygius* species complex and morphologically corresponding to species *L. agilis* (sample BA831) and *L. erythrocephalus/L. schuleri* (samples BA184, BA219, BA736, BA792, BA796, BA797, BA829, and BA830). The rest of the data were taken from Ganske and colleagues^40^.

The morphological dataset contains information on 29 specimens of the species *L. stygius* (s. str.), including both the specimens from the type series, stored in the Naturhistorisches Museum Wien (NHMW), and the newly collected specimens from the Postojna-Planina Cave System, stored at the collection of the Subterranean Biology Lab, Department of Biology, Biotechnical Faculty, University of Ljubljana (SubBioLab). We included nine adults, i.e. maturus-like specimens^75^ out of altogether 12 examined syntypes, stored in 80% ethanol. Two of them were included in the analysis of the arrangement of spurs on the legs, i.e. plectrotaxy. In addition to the historical material, we also included 20 newly collected best preserved adult specimens (10 males, 10 females) stored in 70 or 96% ethanol.

The information on the literature records of *L. stygius*, the specimens used for the molecular analyses and the morphological dataset are available for download as Microsoft Excel (.xlsx) files from Figshare^43^.

### DNA extraction and laboratory protocols

To extract genomic DNA from newly collected specimens included in the molecular dataset we used two extraction kits, GenElute Mammalian Genomic DNA (Sigma-Aldrich, USA) or MagMAX DNA Multi-Sample Kit (Thermo Fisher Scientific, USA). We amplified two mitochondrial (Cytochrome *c* oxidase I—COI, 16S rRNA) and one nuclear (28S rRNA) gene fragments. A list of oligonucleotide primers and polymerase chain reaction (PCR) amplification protocols are given in Supplementary Tables S1–S3 online. The purified amplification products were sent for bidirectional Sanger sequencing to Macrogene Europe laboratory (Amsterdam, Netherlands). We verified, edited, assembled and aligned the sequences in Geneious Prime 2022.2.2 (Biomatters, New Zealand). COI fragments were aligned using MUSCLE^76^, while 16S rRNA and 28S rRNA fragments using MAFFT^77^ and E-INS-i algorithm. We concatenated the single gene alignments into a joint, 2152 base pairs long, COI, 16S rRNA, and 28S rRNA alignment.

### Phylogenetic analyses and species delimitation

We inferred phylogenetic trees based on concatenated alignment with partition-specific settings using two alternative approaches, i) maximum likelihood and ii) Bayesian inference. Maximum likelihood approach was implemented within IQ-TREE^78^, and the final tree was accessed after running 1000 ultrafast bootstrap replicates with IQTREE ver. 2.3.5^79^ ran on a local server. Bayesian inference was implemented using MrBayes ver. 3.2.7^80^, and substitution models as obtained with the locally run IQTREE. Partitions and substitution models are available in Supplementary Information online. We performed two parallel runs, each comprising eight Markov chain Monte Carlo (MCMC), for 30 million generations, with every 500th tree sampled. We discarded 25% of the sampled trees as a burn-in and inferred a 50% majority rule consensus tree using the remaining trees. We checked the convergence of the parallel runs using Tracer ver. 1.7.2^81^. We inferred individual gene trees based on maximum likelihood as described above. Their topologies were visualised in R version 4.3.0^82^ and Rstudio version 2024.9.1.394^83^, using package phytools 2.3.0^84^ and ape 5.8^85^.

To delimit taxonomical units, we used the data subset of (i) COI and (ii) 16 s rRNA sequences from the subgroup containing specimens of the *L. stygius* complex^43^ (Supplementary Figs. S5–S12 online). Species delimitation was run using two alternative approaches, namely the distance-based method Assemble Species by Automatic Partitioning (ASAP)^66^ and the phylogeny-based method Multi-rate Poisson tree processes (mPTP)^67^. The ASAP analysis was done with software ASAPy 1.0, a part of iTaxoTools^86^, using Kimura’s two-parameter substitution model. For COI based delimitation we had to account for potential bias due to overrepresentation of specimens from Planinska jama, we repeated the analysis using three alternative subsets, each including six randomly chosen specimens this site. For the PTP analysis, we collapsed the original dataset to haplotypes using FaBox ver. 1.61^87^, on a web server https://users-birc.au.dk/palle/php/fabox/. The derived dataset was used to infer phylogenetic tree using IQ-TREE and applying a codon specific partition. The resulting phylogenetic trees were used to run mPTP analysis on a web server https://mptp.h-its.org/#/tree. All resulting trees were visualised using FigTree ver. 1.4.4^88^, whereas the maps were produced using QGIS version 3.16 (Hannover).

### Morphological analyses of the species *Lithobius stygius* (s. str.)

We examined, measured and photographed the specimens included in the morphological dataset using: i) Nikon SMZ25 stereomicroscope with mounted Nikon DS-Ri2 camera, using NIS-Elements Microscope Imaging Software (version 5.02) with an Extended Depth of Focus (EDF) patch (Nikon Corporation, Tokyo, Japan); ii) the same model of stereomicroscope and imaging software (version 5.42), but with mounted Digital Sight 10; or iii) Canon EOS 90D digital camera, where stacked images were assembled using Zerene Stacker ver. 1.04 (Zerene Systems LLC, Richland, USA) available for download from https://www.zerenesystems.com/cms/home. Images of the specimens were edited using Adobe Photoshop 2020. We chose measured characters based on commonly used ones in genus *Lithobius* (e.g.^24,89^). All measurements were made by a single person (Anja Kos).

The general morphological terminology follows Bonato and colleagues^90^, whereas the terminology for the ontogenetic stages follows Verhoeff^91^ with modifications by Stojanović and colleagues^75^. The following abbreviations are used: V—ventral, D—dorsal; C—coxa, t—trochanter, P—prefemur, F—femur, T—tibia; a—anterior, m—median, p—posterior; T, TT—tergite, tergites, Po—porodont, TO—Tömösváry’s organ. We recorded the characters on both sides of the body (left and right), where possible.

## Supplementary Information

Below is the link to the electronic supplementary material.

Supplementary Material 1

## Data Availability

Data generated during this study are available for download from the Supplementary Information, Figshare^43^, and the NCBI GenBank database ([https://www.ncbi.nlm.nih.gov/genbank/]) under accession numbers OR144376–OR144377, PV708099–PV708569, and PV708975–PV709249. Data can also be directly requested from the corresponding author, Anja Kos (anja.kos@uni-lj.si).
