# Supplementary material for: Disentangling the cave centipede Lithobius stygius species complex through molecular phylogenetics and redescription of L. stygius s. str
Source: Sci Rep. 2025 Nov 28;15:42687. doi: 10.1038/s41598-025-26880-7 (PMC12663195; doi:10.1038/s41598-025-26880-7)
Supplement: Supplementary file 1 — Supplementary Material 1 [file 41598_2025_26880_MOESM1_ESM.pdf]

Supplementary Information for:

# Disentangling the cave centipede *Lithobius stygius* species complex through molecular phylogenetics and redescription of *L. stygius* s. str.

Anja Kos, Nesrine Akkari, Teo Delić, Ana Komerički, Dalibor Stojanović, Maja Zagmajster

**Content:**

Supplementary information on molecular methods ........................................................................ 2

Supplementary information on the results of phylogenetic analyses ……………………………………………. 3

Supplementary information associated with the taxonomy of the *Lithobius stygius* and its redescription ........................................................................................................................ 17

## Supplementary information on molecular methods

**Supplementary Table 1:** List of oligonucleotide primers used in the study.

| Gene | Primer | Reference |
|---|---|---|
| COI | LCO 1490 – HCO 2198 | [1] |
| COI | LCO 1490 – HCOout | [1,2] |
| 16S rRNA | 16ar – 16br | [3] |
| 28S rRNA | 28lev2 – 28des2 | [4] |

**Supplementary Table 2:** PCR protocols used in the molecular analysis.

| COI | | 16S rRNA | | 28S rRNA | |
|---|---|---|---|---|---|
| 94 °C 3 min | | 94 °C 4 min | | 94 °C 3 min | |
| 95 °C 30 s | | 94 °C 30 s | | 94 °C 45 s | |
| 48 °C 30 s | 35x | 47 °C 30 s | 35x | 94 °C 45 s | 30x |
| 72 °C 1 min | | 72 °C 45s | | 94 °C 45 s | |
| 72 °C 1 min | | 72 °C 5 min | | 94 °C 45 s | |

**Supplementary Table 3:** DNA partitions and partition specific optimal substitution models used for Bayesian phylogenetic inference.

| Gene | Partition | Substitution model |
|---|---|---|
| COI | 1-675\3 | SYM+I+G4 |
| COI | 2-675\3 | HKY+I |
| COI | 3-675\3 | GTR+G4 |
| 16S rRNA | 676-1230 | GTR+I+G4 |
| 28S rRNA | 1231-2154 | GTR+I |

**Supplementary information on the results of phylogenetic analyses**

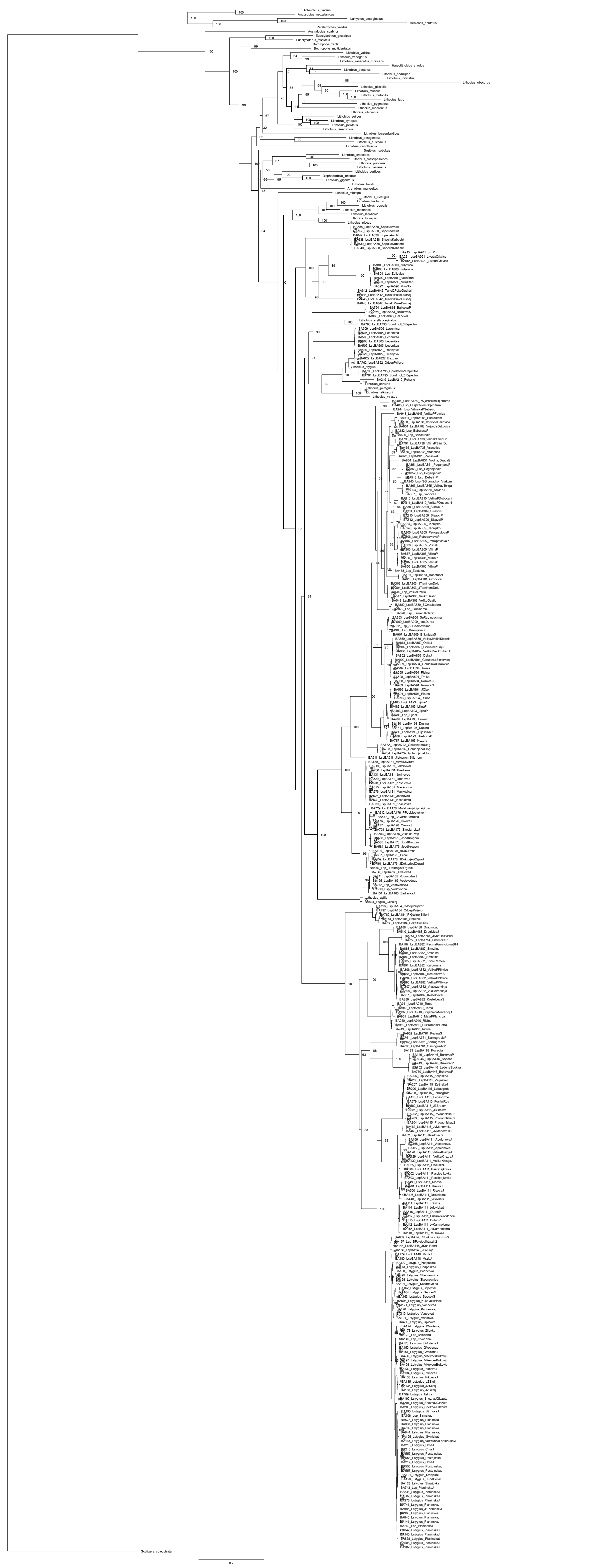

**Supplementary Figure 1:** Maximum likelihood phylogeny of the full molecular dataset based on the concatenated dataset of three genes (COI, 16S rRNA, 28S rRNA).

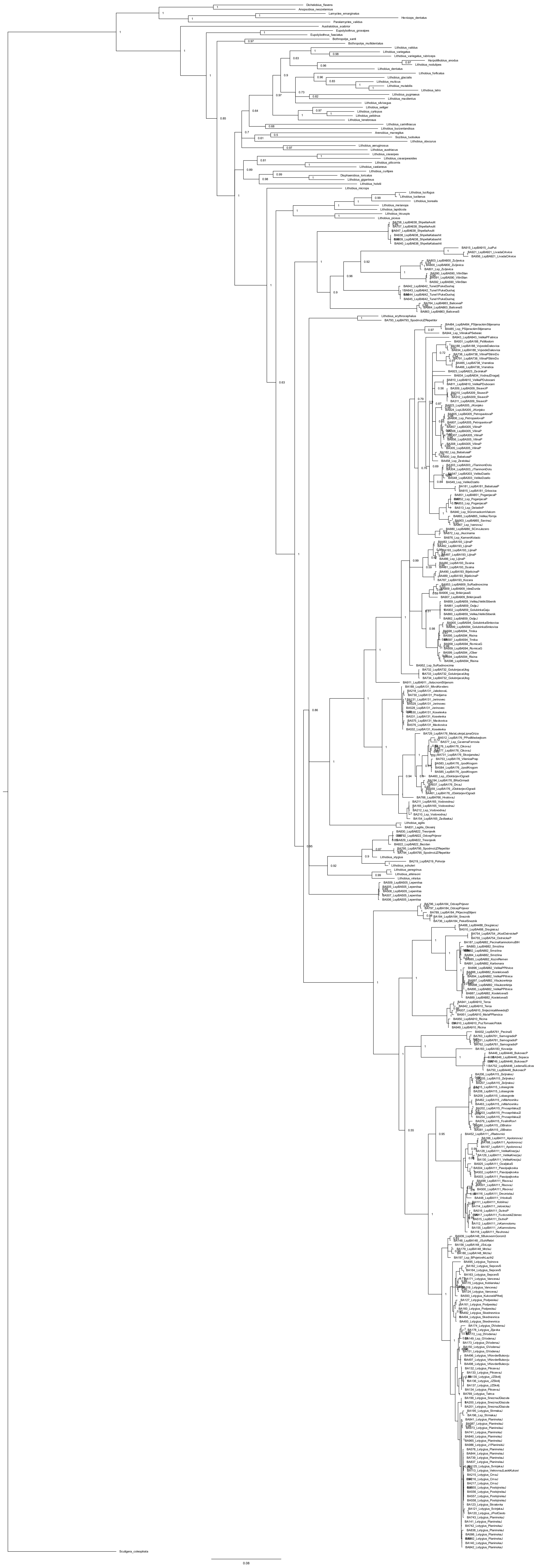

**Supplementary Figure 2:** Bayesian phylogeny of the full molecular dataset based on the concatenated dataset of three genes (COI, 16S rRNA, 28S rRNA).

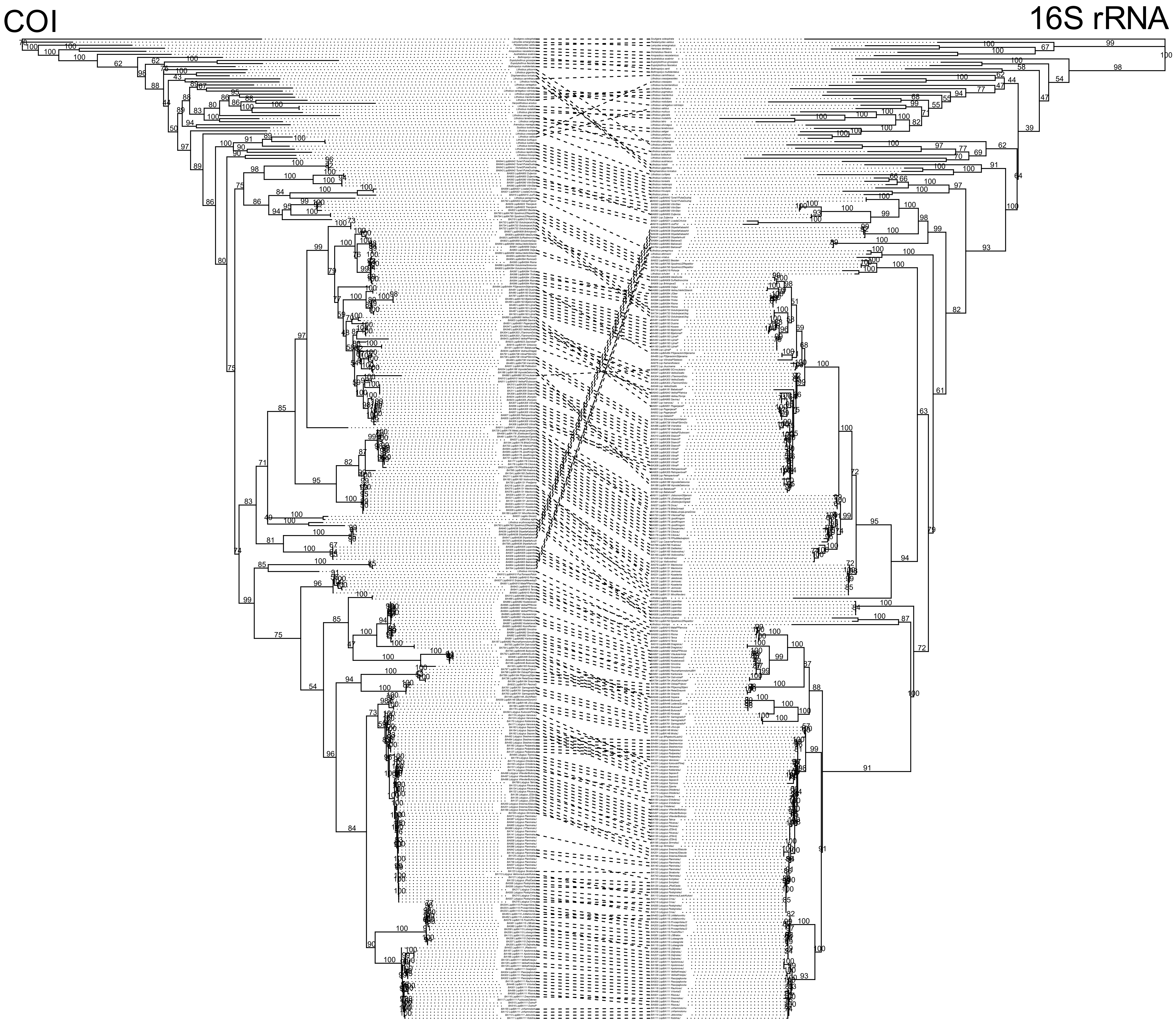


**Supplementary Figure 3:** Comparison of gene-tree topologies between mitochondrial markers COI (left) and 16S rRNA (right).

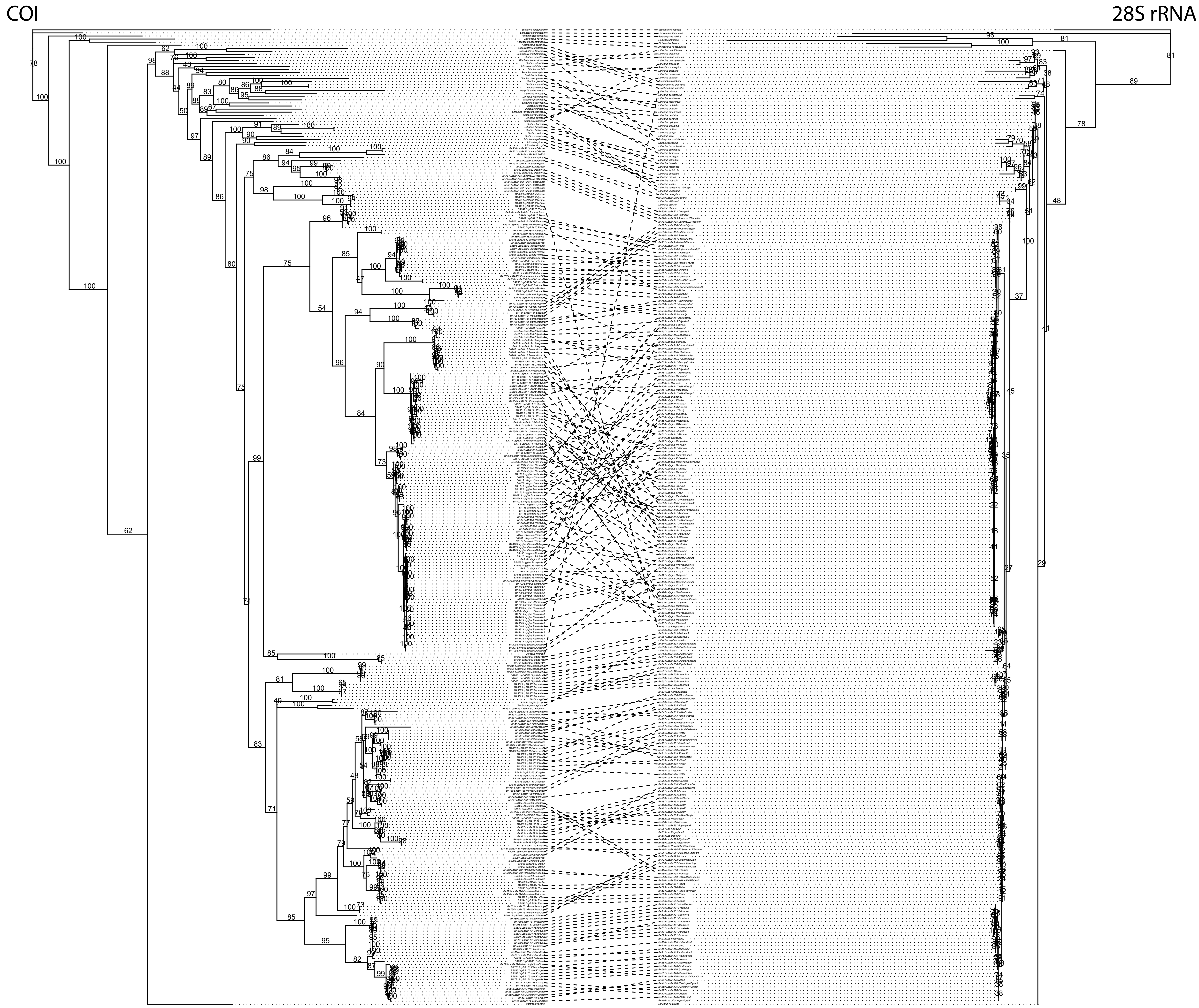


**Supplementary Figure 4:** Comparison of gene-tree topologies between mitochondrial marker COI (left) and nuclear 28S rRNA (right).

```
> 10 Best asap scores (probabilities evaluated with seq length:675)
  distance  #species   #spec w/rec  p-value pente asap-score
*  0.0089      97          97  1.776e-01 4.159777e-03          9.000000
*  0.0119      86          86  2.715e-01 4.505581e-03          9.500000
*  0.0119      83          83  4.651e-01 4.505581e-03          11.000000
*  0.0134      80          80  6.946e-01 4.633723e-03          14.500000
*  0.0388      52          52  1.437e-01 3.169778e-03          16.500000
*  0.0188      68          68  3.633e-01 3.574655e-03          17.000000
*  0.0498      50          50  6.926e-01 3.879665e-03          20.000000
   0.0718      29          29  5.409e-01 3.183329e-03          21.500000
*  0.0112      87          87  6.248e-01 3.283642e-03          21.500000
*  0.0120      82          82  8.323e-01 4.505581e-03          22.000000
```

**Supplementary Figure 5:** Proposed partitions of the ASAP delimitation algorithm of the subset containing COI sequences of monophylum including all newly analyzed samples.

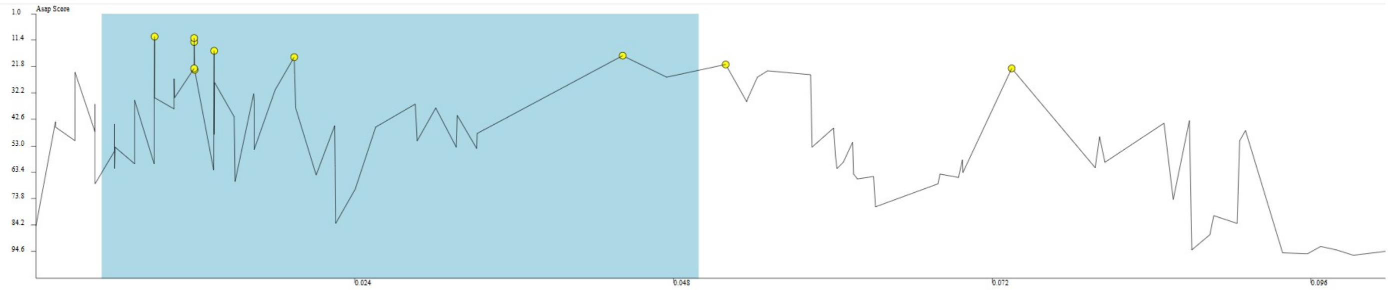


**Supplementary Figure 6:** Results of the ASAP delimitation algorithm of the subset containing COI sequences of monophylum including all newly analyzed samples. Graph shows the relationship of the ASAP score and threshold distance. Yellow dots represent ten best partitions.

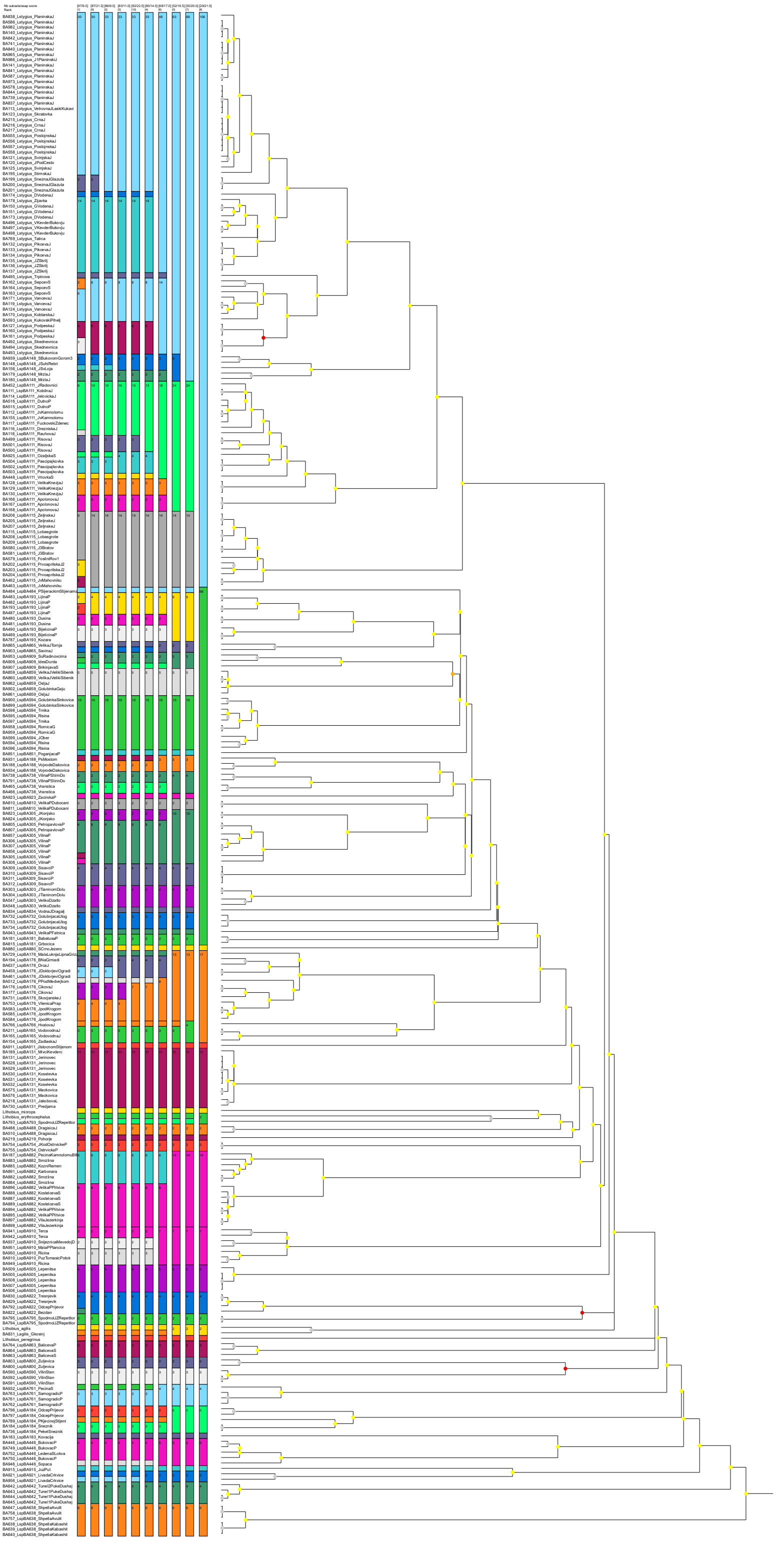

**Supplementary Figure 7:** Proposed groups of the ASAP delimitation algorithm of the subset containing COI sequences of monophylum including all newly analyzed samples.

> 10 Best asap scores (probabilities evaluated with seq length:540)

| | distance | #species | #spec w/rec | p-value | pente | asap-score |
|---|---|---|---|---|---|---|
| * | 0.0401 | 31 | 31 | 5.205e-03 | 2.977347e-03 | 10.500000 |
| * | 0.0060 | 83 | 83 | 4.671e-01 | 3.565389e-03 | 14.500000 |
| | 0.0040 | 88 | 88 | 1.936e-01 | 2.951869e-03 | 16.000000 |
| | 0.0020 | 146 | 146 | 5.903e-02 | 2.832842e-03 | 20.500000 |
| | 0.0020 | 118 | 118 | 1.876e-01 | 2.832842e-03 | 22.000000 |
| * | 0.0080 | 75 | 75 | 7.445e-01 | 3.497040e-03 | 24.500000 |
| | 0.0020 | 139 | 139 | 1.756e-01 | 2.832842e-03 | 25.500000 |
| | 0.0040 | 90 | 90 | 4.351e-01 | 2.951869e-03 | 25.500000 |
| * | 0.0060 | 84 | 84 | 7.924e-01 | 3.565389e-03 | 26.500000 |
| * | 0.0079 | 78 | 78 | 7.705e-01 | 3.497040e-03 | 27.000000 |

**Supplementary Figure 8:** Proposed partitions of the ASAP delimitation algorithm of the subset containing 16S rRNA sequences of monophylum including all newly analyzed samples.

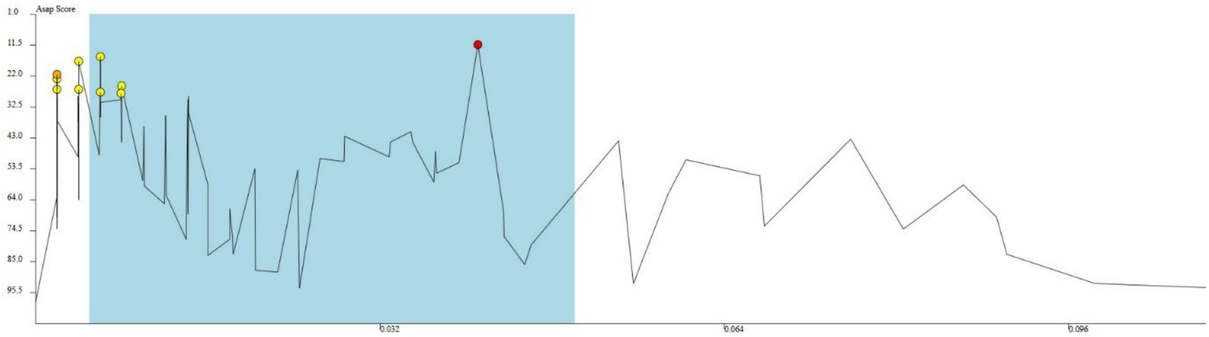


**Supplementary Figure 9:** Results of the ASAP delimitation algorithm of the subset containing 16S rRNA sequences of monophylum including all newly analyzed samples. Graph shows the relationship of the ASAP score and threshold distance. Dots represent ten best partitions.

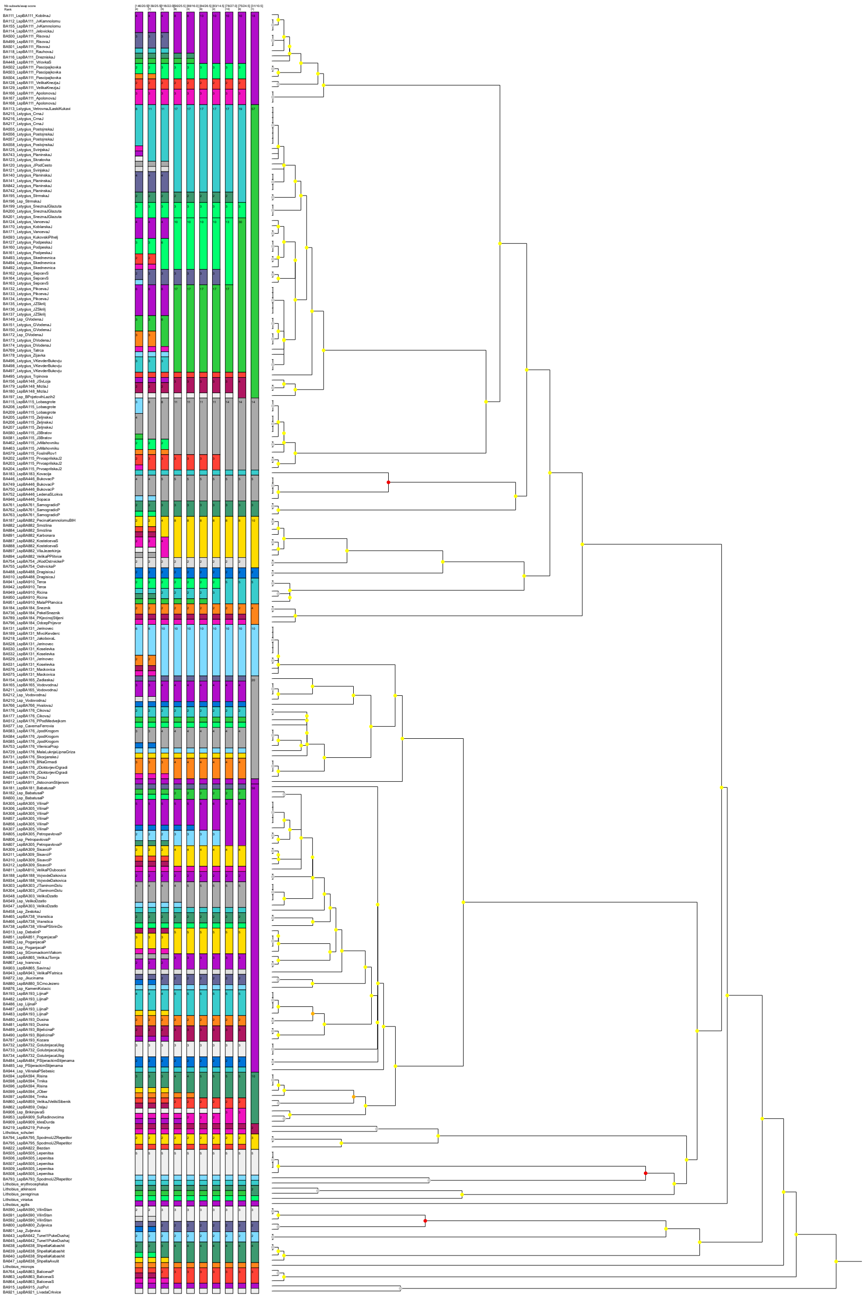

**Supplementary Figure 10:** Proposed groups of the ASAP delimitation algorithm of the subset containing 16S rRNA sequences of monophylum including all newly analyzed samples.

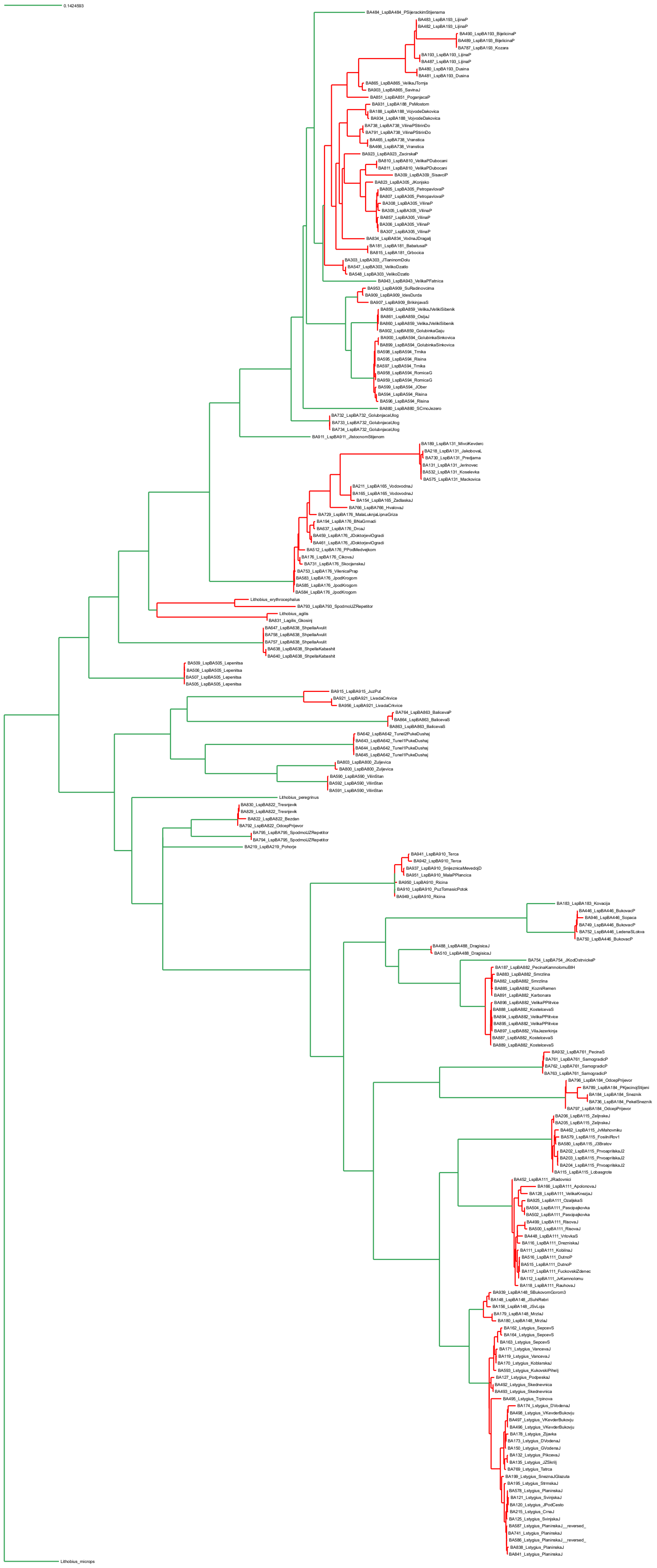

**Supplementary Figure 11:** Resulting phylogenetic tree with delimitation based on the mPTP delimitation algorithm. The input is the subset containing distinct haplotypes of the COI sequences of monophylum including all newly analyzed samples.

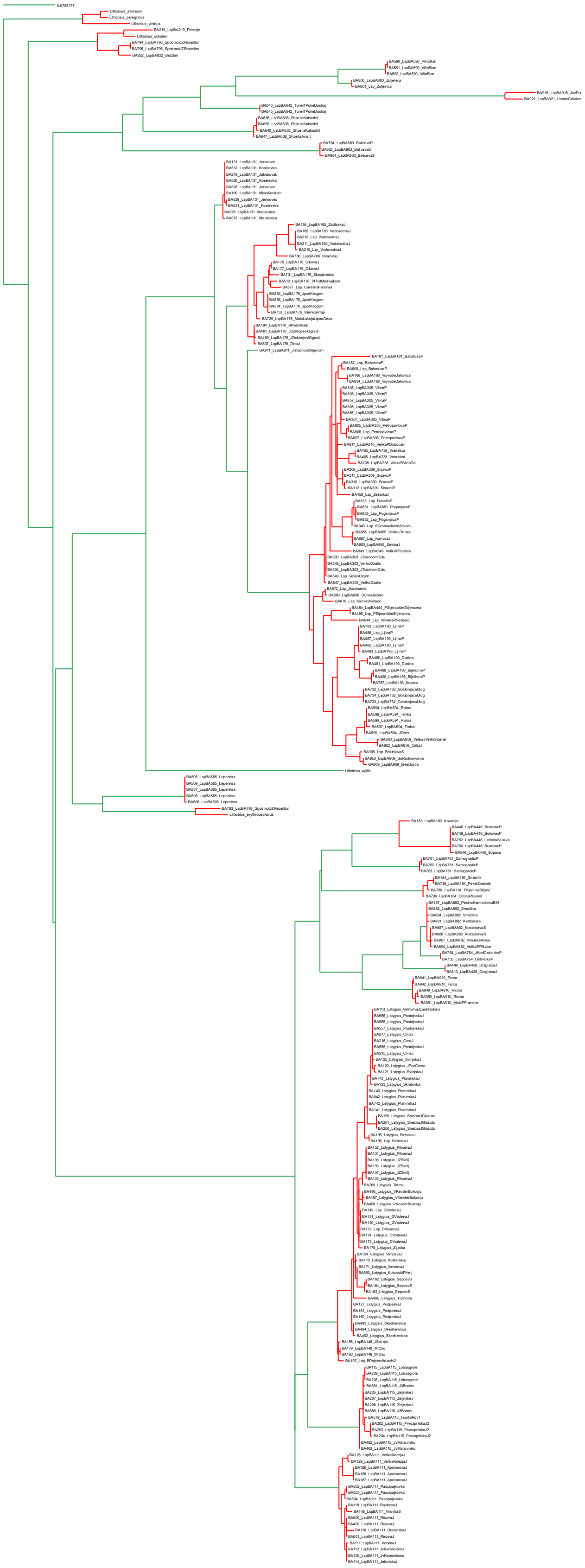

**Supplementary Figure 12:** Resulting phylogenetic tree with delimitation based on the mPTP delimitation algorithm. The input is the subset containing distinct haplotypes of the 16S rRNA sequences of monophylum including all newly analyzed samples.

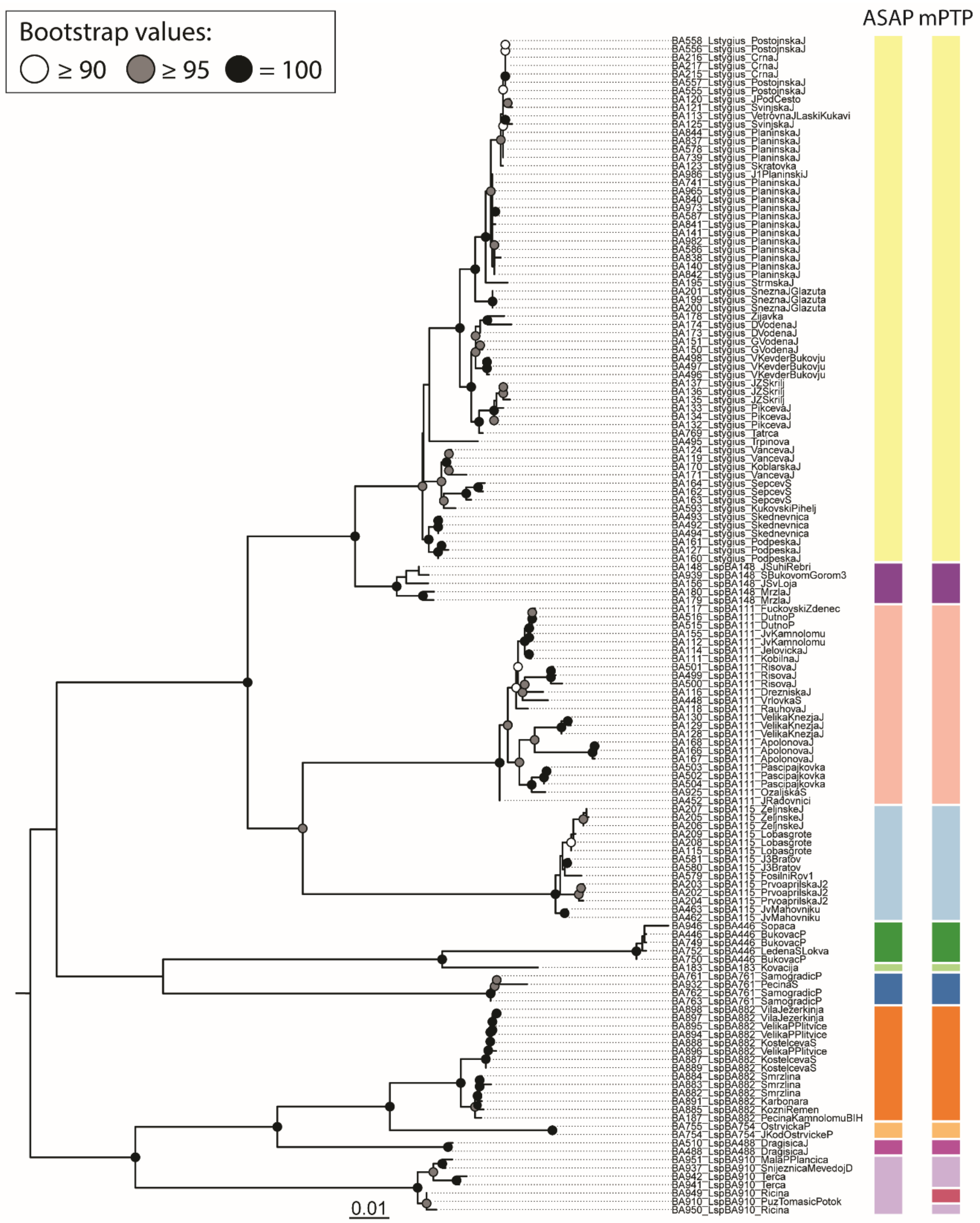


**Supplementary Figure 13:** Phylogenetic relationships within the Clade 1 of *L. stygius* complex (including tip labels) and its delimitation into MOTUs based on COI. Phylogenetic tree was calculated using maximum likelihood approach basing on gene fragments COI, 16S rRNA and 28S rRNA.

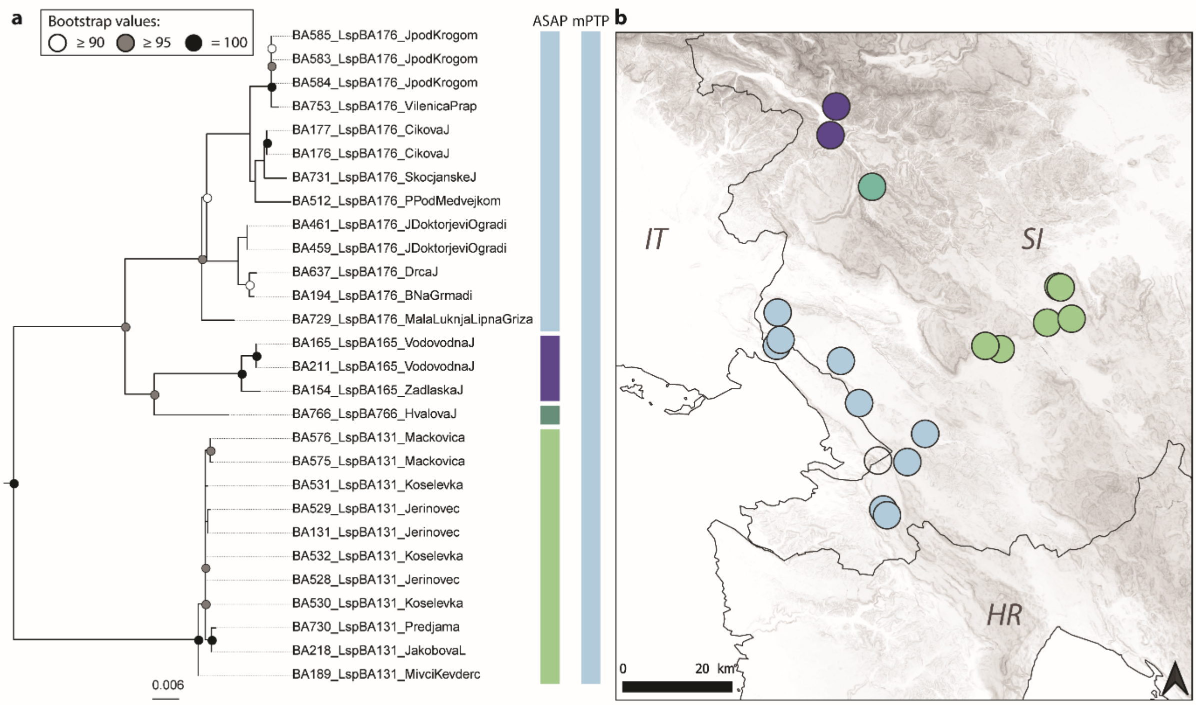


**Supplementary Figure 14:** (**a**) Phylogenetic relationships within the Clade 2 of *L. stygius* complex, its delimitation into MOTUs based on COI, and (**b**) their geographical distribution. Phylogenetic tree was calculated using maximum likelihood approach basing on gene fragments COI, 16S rRNA and 28S rRNA. On the map, colours match ASAP delimitation results and empty circles represent samples without assigned MOTU.

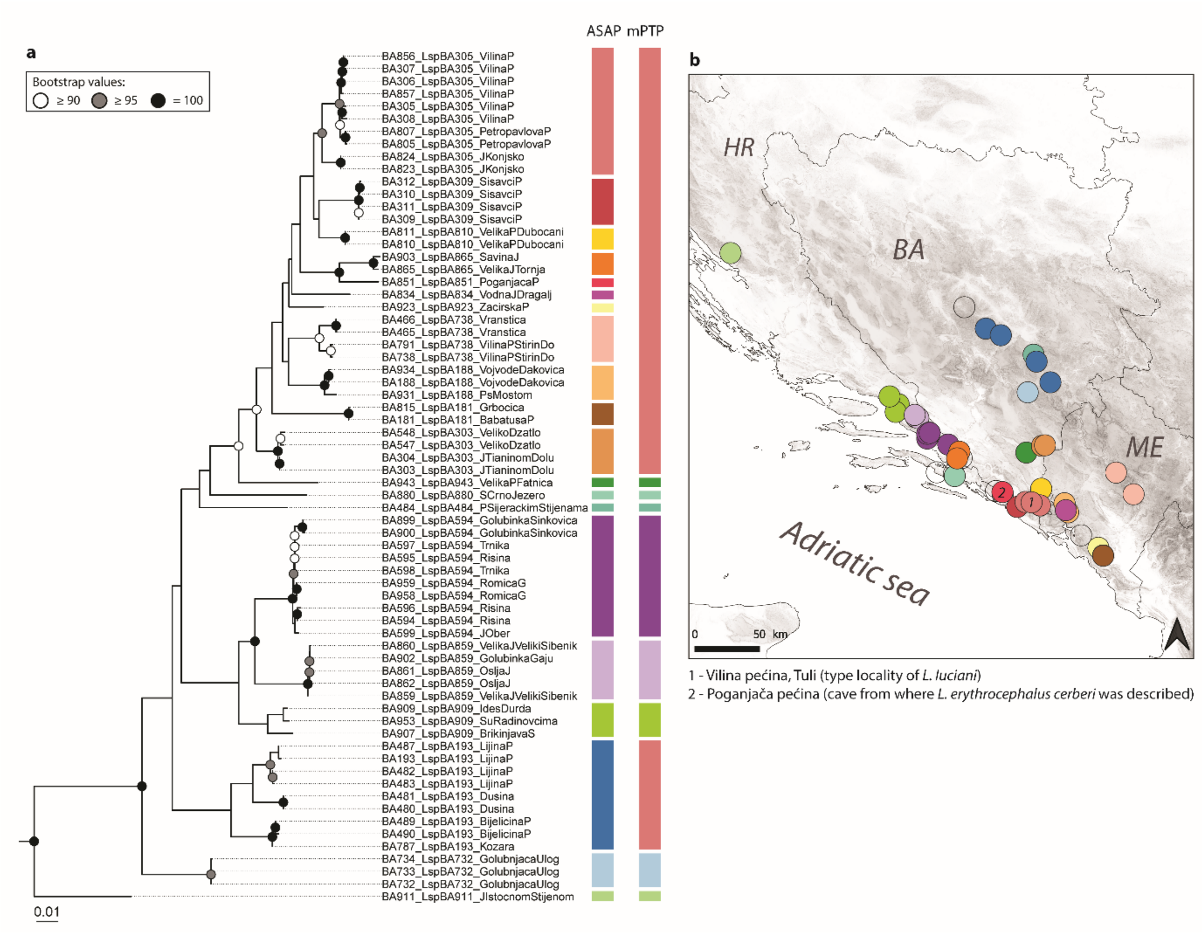


**Supplementary Figure 15:** (**a**) Phylogenetic relationships within the Clade 3 of *L. stygius* complex, its delimitation into MOTUs based on COI, and (**b**) their geographical distribution. Phylogenetic tree was calculated using maximum likelihood approach basing on gene fragments COI, 16S rRNA and 28S rRNA. On the map, colours match ASAP delimitation results and empty circles represent samples without assigned MOTU.

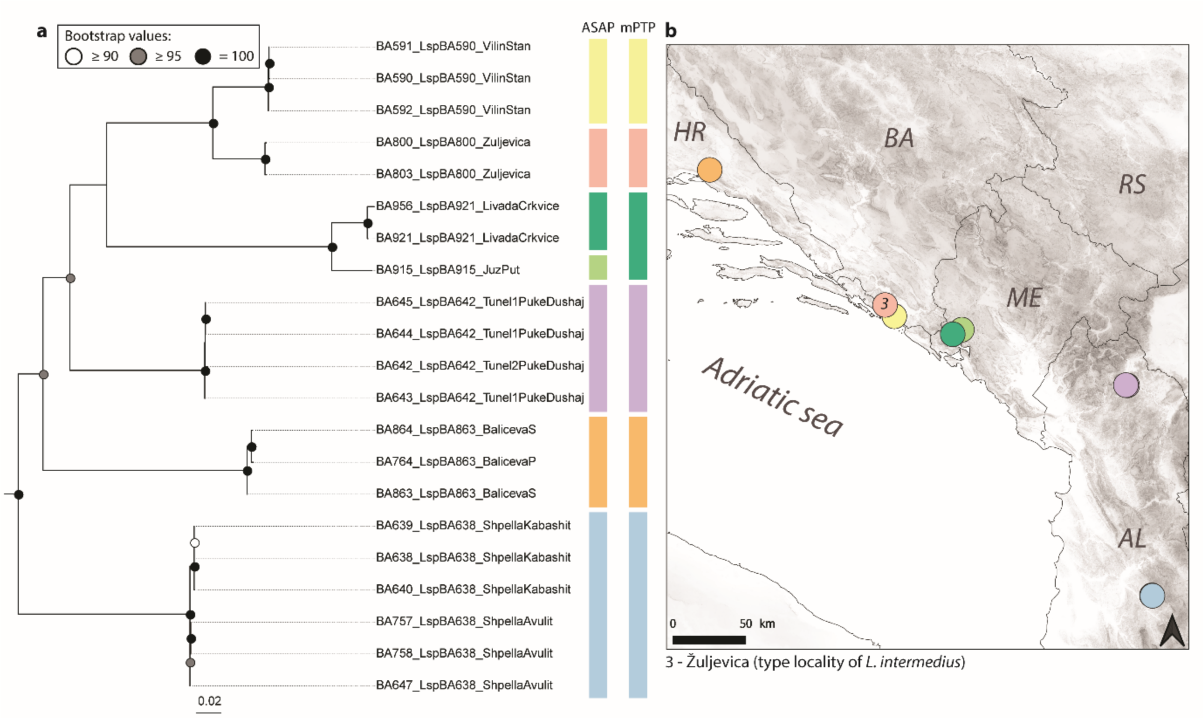


**Supplementary Figure 16:** (**a**) Phylogenetic relationships within the Clade 5 of *L. stygius* complex, its delimitation into MOTUs based on COI, and (**b**) their geographical distribution. Phylogenetic tree was calculated using maximum likelihood approach basing on gene fragments COI, 16S rRNA and 28S rRNA. On the map, colours match ASAP delimitation results.

**Supplementary information associated with the taxonomy of the *Lithobius stygius* and its redescription**

**Supplementary Table 4:** Taxonomic information regarding *Lithobius stygius*.

| Taxonomic name | Previous status | Citation | Status proposed in this study |
|---|---|---|---|
| *Oligobothrus luciani* Folkmanová, 1935 | synonym of *L. stygius* | [5] | valid species *Lithobius luciani* (Folkmanová, 1935) |
| *Lithobius erythrocephalus cerberi* Verhoeff, 1943 | synonym of *L. stygius* | [5] | synonym of *Lithobius luciani* (Folkmanová, 1935) |
| *Lithobius stygius intermedius* Folkmanová, 1946 | synonym of *L. stygius* | [6] | valid species *Lithobius intermedius* Folkmanová, 1946 |
| *Lithobius stygius mazerollensis* Verhoeff, 1937 | subspecies of *L. stygius* | [7] | cannot be reliably addressed, possibly closely related to *L. illyricus* |
| *Lithobius temnensis* Verhoeff, 1943 | possible synonym of *L. stygius* | [5] | not addressed |
| *Lithobius jugoslavicus* Matic & Darabantu, 1968 | possible synonym of *L. stygius* | [5] | not addressed (note: its substitute name is *Lithobius corneliae* Stoev, 1997) |

**Supplementary Table 5:** Differential characters of *Lithobius stygius* in comparison to other similar species.

| Character\Species | *L. stygius* | *L. erythrocephalus* | *L. schuleri* | *L. illyricus* | *L. luciani* | *L. intermedius* |
|---|---|---|---|---|---|---|
| **Ocelli** | 6–7, in two or three rows | 10–14 | 10–14 | 11–13, in three rows | 7, in two rows | 9–10 |
| **Tömösváry's organ** | larger than seriate ocelli | smaller than the largest ocellus in the group | smaller than the largest ocellus in the group | NA | NA | NA |
| **Number of antennal articles** | 33–44 (most commonly 36–37) | 28–35 | 28–35 | 50–58 | 36 | 50–52 |
| **Female gonopod spurs** | 2+2 elongate spurs | 2+2 spurs (about three times longer than broad) | 2+2 more elongate spurs (four to five times longer than broad) | 2+2 very elongate spurs | NA | NA |
| **Female gonopod claw** | tripartite | tripartite | tripartite | tripartite | NA | NA |
| **VaC spine on Leg 15** | present | present | present | present | absent | present (although not explicitly stated) |
| **Legs of adult male legs** | without modification | 15th tibiae of male dorsoventrally flattened | without modification (almost perfectly cylindrical) | without modification | NA | NA |
| **Reference** | this study | [8] | [8] | [9] | [10] | [11] |

**List of citations of *Lithobius stygius:***

Citations that at least in part most likely refer to MOTU *L. stygius*:

***Lithobius stygius* Latzel, 1880:** Attems, 1949[12]: p. 113; Attems, 1959[13]: p. 295 (tab. 1) *(as Lithobius stygius stygius);* Kos, 1933[14]: p. 18; Kos *et al.*, 2023[15]: p. 166, figs. 3–4, tab. 1; Kos, 1992[16]: p. 357 (tab. 1); Latzel, 1880[9]: p. 113; Manfredi, 1932a[17]: p. 83; Manfredi, 1932b[18]: p. 7; Manfredi, 193[19]5: p. 256; Manfredi, 1936[20]: p. 80; Manfredi, 1940[21]: p. 227; Matic & Darabantu, 1968[22]: p. 216; Matic & Stentzer, 1977[23]: p. 56, figs. 1–5, tabs. 1–2; Matic, 1978[24]: p. 166; Matic, 1979[25]: p. 154; Stagl & Zapparoli, 2006[26]: p. 34; Stoev, 1997[6]: p. 92 *(*as *Lithobius stygius* and *Lithobius stygis stygis* Latzel, 1880), tab. 1 (*L. stygius*); Verhoeff, 1900[27]: p. 159; Verhoeff, 1937[7]: p. 212; Wolf, 1934–38[28]: p. 530; Zagmajster *et al.*, 2021[29]: p. 7, tab. 1; Zapparoli, 1989[30]: p. 575

***Archilithobius stygius* (Latzel, 1880):** Attems, 1929[31]: p. 302

Other citations, potentially referring to other similar species:

***Lithobius stygius* Latzel, 1880:** Anonimus, 1955[32]: p. 25; Beron *et al*., 2011[33]: p. 605; Dobroruka, 1965[34]: p. 399, fig. 4; Ganske *et al*., 2021[35]: p. 166, figs. 5, 7–9, tab. 1; Kovačević, 1918[36]: p. 73; Kovačević, 1931[37]: p. 67; Langhoffer, 1912[38]: p. 362; Langhoffer, 1915a[39]: p. 18; Langhoffer, 1915b[40]: p. 65; Pavlova, 2009[41]: p. 109 (tab. 2); Polak & Pipan, 2021[42]: p. 9; Stoev, 2001a[5]: p. 109; Stoev, 2001b[43]: p. 31, fig. 2, tabs. 4–5, 12; Stoev, 2002[44]: p. 7, tabs. 5–6; Vagalinski & Stoev, 2011[45]: p. 132, fig. 3, tabs. 1–2; Verhoeff, 1929[46]: p. 42

**Material examined as part of morphological redescription of *L. stygius***

**Lectotype:** SLOVENIA ● 1 ♀ maturus; lectotype here designated; "Slovenia, Krain, Postojna jama "Adelsberger Grotte" [Postojnska jama]; 45.78272°N, 14.20366°E; leg. Latzel; NHMW MY2085

**Paralectotypes:** SLOVENIA ● 3 ♂♂ maturus, 3 ♀♀ pseudomaturus, 2 ♀♀ praematurus, 1 ♀ immaturus, 1 specimen with removed gonopods; "Slovenia, Krain, Postojna jama "Adelsberger Grotte"" [Postojnska jama]; 45.78272°N, 14.20366°E; leg. Latzel; NHMW MY10656 ● 1 ♂ maturus; "Slovenia, Carniola, Planinska" [Planinska jama]; 45.81990°N, 14.24567°E; leg. Ferdinand J. Schmidt; NHMW MY4049

**Non-type material:** SLOVENIA ● 1 ♂ maturus; Unec, Planina, Planinska jama; 45.81990°N, 14.24567°E; 14. 07. 2020; leg. M. Zagmajster; SubBioLab BA141 ● 1 ♂ maturus; same locality; 29. 10. 2022; leg. M. Zagmajster, J. Bedek, A. Kos, L. Kauf; SubBioLab BA837 ● 1 ♀ maturus; same data as for preceding; SubBioLab BA578 ● 2 ♀♀ maturus; same locality; 30. 06. 2021; leg. Unknown; SubBioLab BA586, BA587 ● 2 ♀♀ maturus; same locality; 8. 06. 2023; leg. B. Rexhepi, A. Kos; SubBioLab BA739, BA741 ● 1 ♀ maturus; same locality; 6. 09. 2005; leg. S. Polak; SubBioLab BA742 ● 1 ♂ pseudomaturus; same data as for preceding; SubBioLab BA743 ● 3 ♂♂ maturus; same locality; 27. 12. 2023; leg. A. Kos; SubBioLab BA838, BA842, BA844 ● 2 ♀♀ maturus; same data as for preceding; SubBioLab BA840, BA841● 1 ♂ maturus; same locality; 15. 05. 2024; leg. A. Kos; SubBioLab BA965 ● 1 ♀ maturus; same data as for preceding; BA973 ● 1 ♂ maturus; Postojna, Postojnska jama; 45.78272°N, 14.20366°E; 20. 10. 2022; leg. A. Kos; SubBioLab BA557 ● 1 ♀ maturus; Postojna, Črna jama; 45.79957°N, 14.20728°E; 16. 08.

2020; leg. P. Gnezda; SubBioLab BA215; ● 1 ♂ maturus; Unec, Planina, Jama 1 pri Planinski jami; 45.82217°N, 14.24747°E; 15. 05. 2024; leg. A. Kos; BA986

***Description of the female BA841 from Planinska jama:***

***Body:*** Fairly robust and evenly broad (Supplementary Fig. 11a). Body length: 15.6 mm. Mid-body width: 1.6 mm.

***Colour:*** Trunk brown with darker median line on tergites (Supplementary Fig. 11a). Distal part of legs and antennae lighter and more yellowish. Head orange-brown with dark spots around ocelli.

***Antennae:*** Long (0.44 of the body length) and thin, when stretched reaching posterior part of T6 (Fig. Supplementary Fig. 11a). Composed of 35–37 antennal articles. Ultimate article more than twice as long as penultimate.

***Cephalic plate:*** Posterior margin straight. Cephalic plate slightly narrowing anteriorly, almost as long as broad (ratio length/width is 0.92:1) (Supplementary Fig. 11a, b). Transverse suture distinctively U-shaped, positioned at anterior half of the cephalic capsule.

***Ocelli:*** Dark violet-brown, round. 1+3, 2 (right) and 1+3, 2, 1 (left). Posterior ocellus is the largest and oval (Supplementary Fig. 11e, f).

***Tömösváry's organ:*** Ovoid (Supplementary Fig. 11e, f). Larger than seriate ocelli, of the same size as posterior ocellus. Lying anteroventral to ocelli on anterolateral margin of cephalic capsule.

***Forcipular* coxosternite*:*** Anterior margin moderately wide, with outer sides slightly higher than inner sides, with 2+2 stout triangular teeth of similar size (Supplementary Fig. 11c, d). Median diastema moderately shallow and wide, V-shaped. Porodont slightly stouter than setae, positioned in front of the lateral edge of outer tooth. Lateral margins of forcipular coxosternite sloping gradually backwards without shoulders.

***Tergites:*** T1 with narrower posterolateral side (Supplementary Fig. 11b). T10 widest of all tergites (Supplementary Fig. 11a). Posterior border of T8 and T10 feebly concave; posterior corner of tergites T9 and T11 obtuse, minute triangular projections are present on T13 (Supplementary Fig. 12a).

***Legs:*** Moderately long. Size progressively increasing toward posterior part, 15$^{th}$ leg reaching 0.42 of the body length. Tarsal articulations in all walking-legs distinct. All apically legs with moderately long and curved claws. Ultimate and penultimate legs with accessory claw. Pores of telopodal glands on inner side in penultimate and ultimate legs present. Plectrotaxy as in Supplementary Table 6.

***Coxal pores:*** 3555/4555 separated from one another by less than their own diameter, moderately large. Round to slightly oval (Supplementary Fig. 12b).

***Female gonopods:*** 2+2 slender acuminate spurs slightly expanded subapically, inner spur a bit smaller than outer (Supplementary Fig. 12b, c). Outer spurs 3.5–4 times and inner spurs 3–3.5 times longer than broad. Tripartite claw with well separated and pointy teeth of similar size. First genital sternite with seven long ventral setae in two rows. Second genital sternite with six ventral long setae and 4–5 (left-right, respectively) medium long and less stout dorsolateral setae. Third genital sternite with two long ventral setae and two smaller less stout dorsolateral setae. No dorsomedial setae.

***Male sexual characters of specimen BA965 from Planinska jama:*** First genital sternite with several long ventral setae and concave posterior margin. Gonopod uniarticulated, short, with two long setae (Supplementary Fig. 12d). Legs 14 and 15 without any modifications.

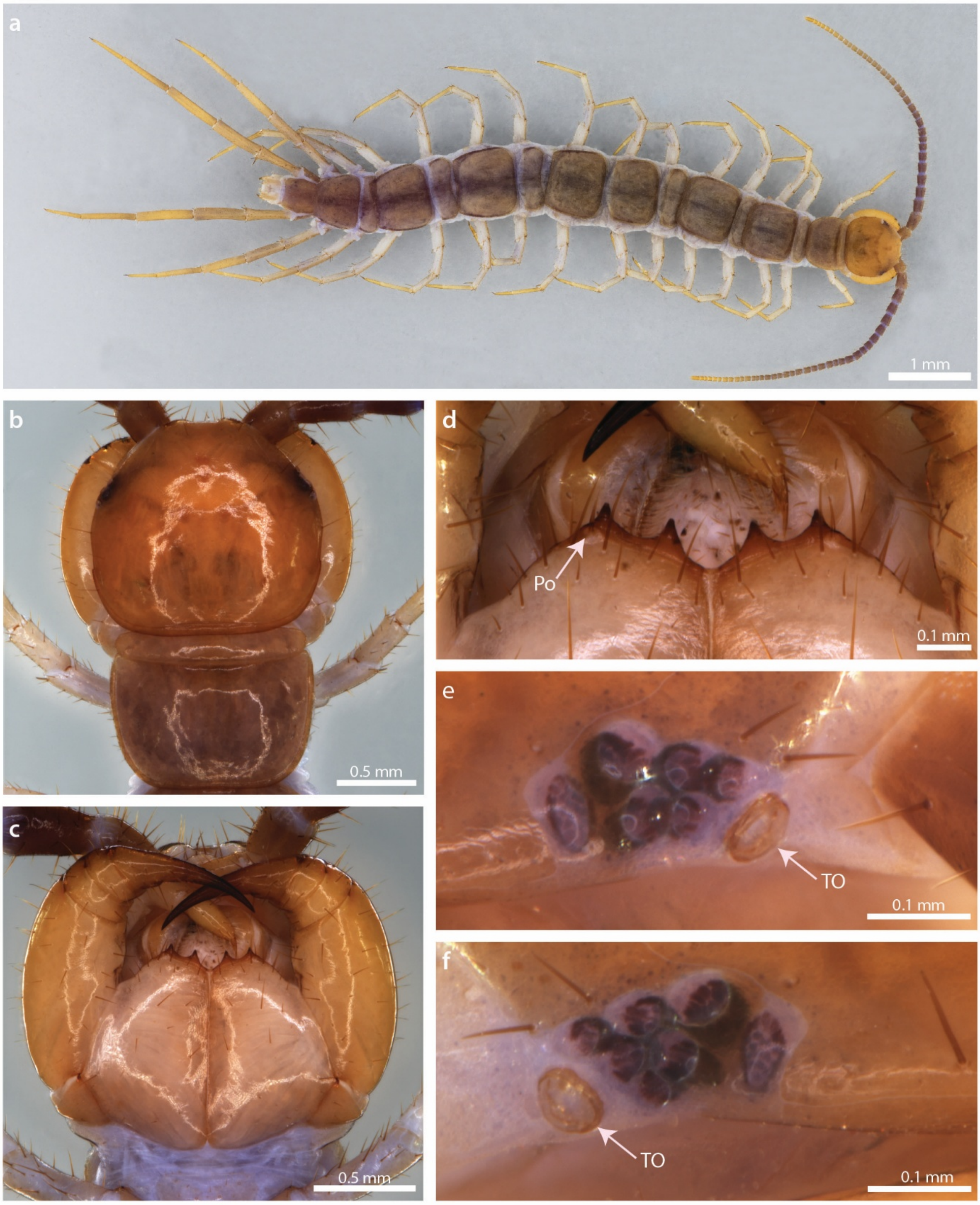


**Supplementary Figure 17:** *Lithobius stygius* female BA841 from Planinska jama. (**a**) Habitus, dorsal view; (**b**) cephalic plate and T1, dorsal view; (**c**) forcipular segment, ventral view; (**d**) close-up of anterior margin of the forcipular coxosternite, ventral view; (**e**) ocellar area and Tömösváry's organ, lateral view, right side; (**f**) ocellar area and Tömösváry's organ, lateral view, left side. Abbreviations: Po — porodont, TO — Tömösváry's organ.

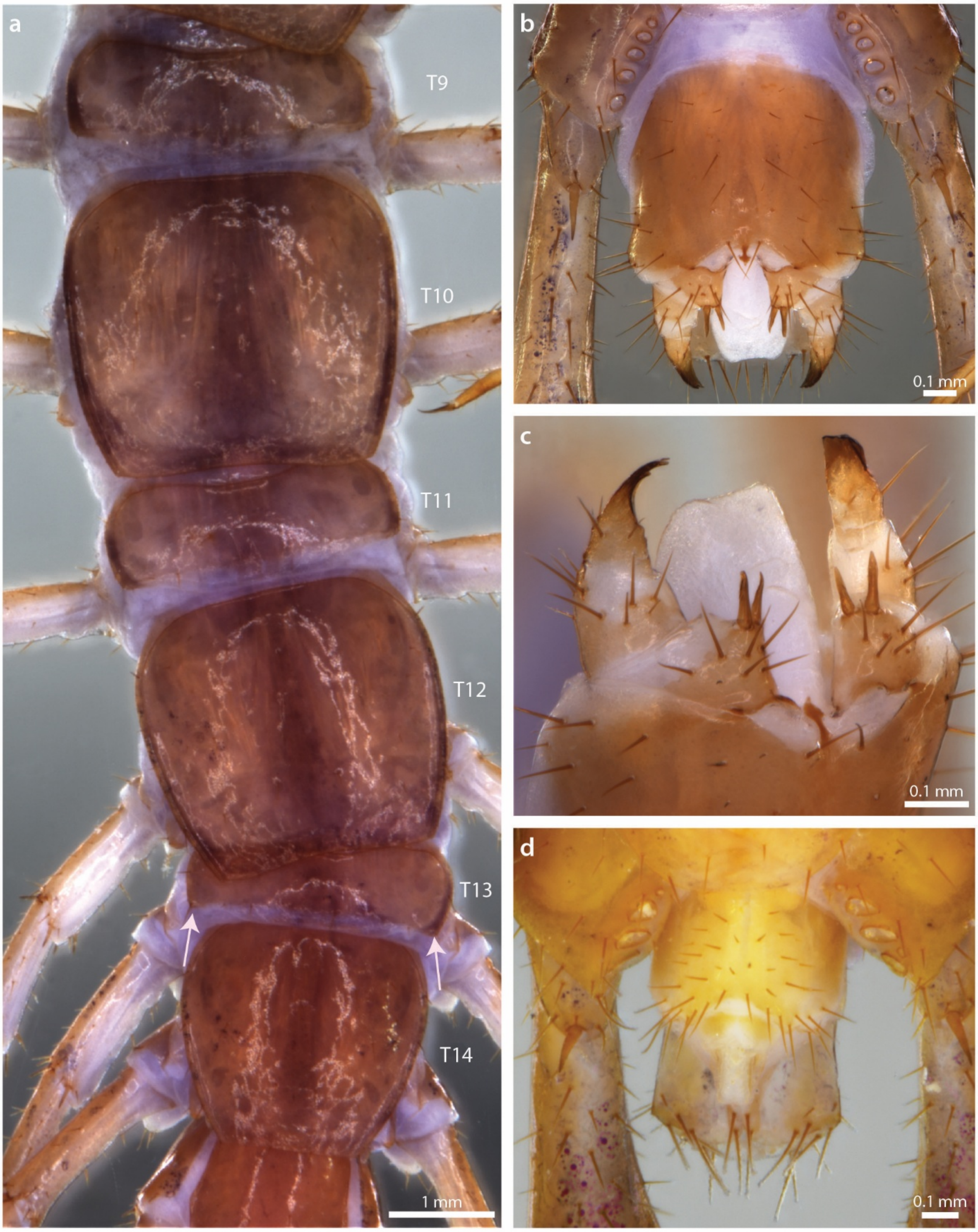


**Supplementary Figure 18:** *Lithobius stygius* female BA841 (**a–c**) and male BA965 (**d**) from Planinska jama. (**a**) TT9–13, dorsal view; (**b**) posterior segments and gonopods, ventral view; (**c**) close-up of gonopods, ventrolateral view; (**d**) posterior segments and gonopods, ventral view. Abbreviations: T — tergite. Arrows point to minute projections.

**Supplementary Table 6:** Plectrotaxy of *Lithobius stygius*, female BA841.

| Leg pair | Ventral | | | | | Dorsal | | | | |
|---|---|---|---|---|---|---|---|---|---|---|
| | **C** | **t** | **P** | **F** | **T** | **C** | **t** | **P** | **F** | **T** |
| **1** | / | / | / | / | m | / | / | p | a | a |
| **2** | / | / | / | am | m | / | / | mp | ap | a(p) |
| **3** | / | / | (p) | am | m | / | / | mp | ap | ap |
| **4** | / | / | p | am | m | / | / | mp | ap | ap |
| **5** | / | / | p | am | m | / | / | mp | ap | ap |
| **6** | / | / | p | am | m | / | / | mp | ap | ap |
| **7** | / | / | p | am | m | / | / | mp | ap | ap |
| **8** | / | / | p | am(p) | m | / | / | mp | ap | ap |
| **9** | / | / | (m)p | am | (a)m | / | / | mp | ap | ap |
| **10** | / | / | mp | amp | (a)m | / | / | amp | ap | ap |
| **11** | / | / | mp | amp | (a)m | / | / | amp | ap | ap |
| **12** | / | / | (a)mp | amp | am | / | / | amp | ap | ap |
| **13** | / | (m) | amp | amp | am | a | / | amp | ap | ap |
| **14** | (a) | m | amp | amp | am | a | / | amp | p | p |
| **15** | a | m | amp | amp | / | a | / | amp | p | / |

## References:


1. Folmer, O., Black, M., Hoeh, W., Lutz, R. & Vrijenhoek, R. DNA primers for amplification of mitochondrial cytochrome c oxidase subunit I from diverse metazoan invertebrates. *Mol Mar Biol Biotechnol.* **3**(5), 294–299 (1994).
2. Carpenter, J. M. Towards simultaneous analysis of morphological and molecular data in Hymenoptera. *Zool. Scr.* **28**(1-2), 251–260 (1999).
3. Simon, C., Franke, A., & Martin, A. The polymerase chain reaction: DNA extraction and amplification in *Molecular techniques in taxonomy* (Hewitt, G. M., Johnston, A. W. B., & Young, J. P. W.), 329–355 (Springer Berlin Heidelber, 1991).
4. Verovnik, R., Sket, B., & Trontelj, P. The colonization of Europe by the freshwater crustacean *Asellus aquaticus* (Crustacea: Isopoda) proceeded from ancient refugia and was directed by habitat connectivity. *Mol. Ecol.* **14**(14), 4355–4369 (2005).
5. Stoev, P. A synopsis of the Bulgarian cave centipedes (Chilopoda). *Arthropoda Sel.* **10**, 31–54 (2001a).
6. Stoev, P. A check-list of the centipedes of the Balkan peninsula with some taxonomic notes and a complete bibliography (Chilopoda). *Entomol. Scand.* **51**, 87–105 (1997).
7. Verhoeff, K. W. Chilopoden-Studien. Zur Kenntnis der Lithobiiden. *Arch.Naturgesch* **6**, 171–257 (1937).
8. Dányi, L. On the occurrence of *Lithobius erythrocephalus* CL Koch, 1847, and *Lithobius schuleri* Verhoeff, 1925 (Myriapoda: Chilopoda) in Hungary. *Folia Hist.-Nat. Mus. Matraensis* **30**, 105–113 (2006).
9. Latzel, R. *Die Myriapoden der Österreichisch-Ungarishen Monarchie, I. Chilopoden*. 1–288 (A. Holder, 1880).

10. Folkmanová, B. Nov druhy stonozek celedi Lithobiidae z balkánskych jeskyn. *Priroda* **28**, 172–176 (1935).
11. Folkmanová, B. Noví Lithobiové (Chilopoda) z balkánských jeskyň. *Priroda* **38**, 57–70 (1946).
12. Attems, C. G. Die Myriopodenfauna der Ostalpen. *Sitz. Oesterr. Ak. Wiss. Wien* **158**, 79–153 (1949).
13. Attems, C. G. Die Myriopoden der Höhlen der Balkan-Halbinsel. *Ann. Naturhist. Mus. Wien* **63**, 281–406 (1959).
14. Kos, F. Zoološki oddelek in *Vodnik po zbirkah Narodnega muzeja v Ljubljani. Prirodopisni del*. 7–118 (Narodni muzej, 1933).
15. Kos, A. *et al.* The overview of lithobiomorph centipedes (Chilopoda, Lithobiomorpha) from caves of Slovenia. *Subterr. Biol.* **45**, 165–185 (2023).
16. Kos, I. A Review of the Taxonomy, Geographical Distribution and Ecology of the Centipedes of Yugoslavia. *Ber. Naturwiss.-med. Ver. Innsb* **10**, 353–360 (1992).
17. Manfredi, P. Contributo alla conoscenza della fauna cavernicola italiana. *Natura* **23**, 71–96 (1932a).
18. Manfredi, P. I Miriapodi cavernicoli italiani. *Le Grotte d'Italia* **6**, 13–21 (1932b).
19. Manfredi, P. V. contributo alla conoscenza dei Miriapodi cavernicoli Italiani. *Atti Soc. ital. sci. nat., Mus. civ. stor. nat. Milano* **74**, 253–283 (1935).
20. Manfredi, P. II elenco di Miriapodi cavernicoli italiani. Grotte d'Italia **1**, 77–84 (1936).
21. Manfredi, P. VI. contributo alla conoscenza dei Miriapodi cavernicoli Italiani. *Atti Soc. Ital. Sci. Nat. Mus. Civico Storia Nat. Milano* **79**, 221–252 (1940).
22. Matic, Z. & Dărăbanţu, C. Contributions à la connaissance des chilopodes de Yougoslavie. *Razpr. Slov. Akad. Znan. Umetn., Razr. Nar. Vede* **11**, 201–229 (1968).
23. Matic, Z. & Stentzer, I. Beitrag zur Kenntnis der Hundertfüssler (Chilopoda) aus Slowenien. *Biološki Vestn.* **25**, 55–62 (1977).
24. Matic, Z. Chilopodi d'Italia e di Jugoslavia raccolti dal dr. Maurizio Paoletti. *Boll. Soc. entomol. ital.* **110**, 164–166 (1978).
25. Matic, Z. Nouveautes sur la faune des Chilopodes de Yougoslavie. *Biološki Vestn.* **27**, 147–155 (1979).
26. Stagl, V. & Zapparoli, M. *Type specimens of the Lithobiomorpha (Chilopoda) in the Natural History Museum in Vienna*. 1–49 (Verlag des Naturhistorischen Museums, 2006).
27. Verhoeff, K. W. Beitrage zur Kenntniss paläarctischer Myriopoden. XV. Aufsatz: Lithobiiden aus Bosnien, Herzogovina und Dalmatien. *Berliner Entomologische Zeitschrift* **45**, 153–179 (1900).
28. Wolf, B. *Animalium Cavernarum Catalogus, I-III*. 1–1642 (W. Junk's Gravenhage, 1934–38).
29. Zagmajster, M., Polak, S., & Fišer, C. Postojna-Planina cave system in Slovenia, a hotspot of subterranean biodiversity and a cradle of speleobiology. *Diversity* **13**, 271 (2021).
30. Zapparoli, M. I Chilopodi delle Alpi sud-orientali. *Biogeogr*. **13**, 553–584 (1989).
31. Attems, C. G. Die Myriopodenfauna von Albanien und Jugoslavien. *Zool. Jb. Syst.* **56**, 270–306 (1929).
32. Anonimus. Katastar osnovnih podataka o pećinama, jamama in ponorima, te izvršenim istraživanjima. *Speleolog* **3**, 25–28 (1955).
33. Beron, P., Petrov, B. & Stoev, P. Invertebrate cave fauna of the Western Rhodopes (Bulgaria and Greece) in *Biodiversity of Bulgaria 4. Biodiversity of Western Rhodopes (Bulgaria and Greece) II* (ed. Beron, P.) 583–661 (Pensoft & Nat. Mus. Natur. Hist., 2011).
34. Dobroruka, L. J. Ein Beitrag zur Landtierwelt von Korfu. Chilopoda. *Sitzungsber. Kaiserl. Akad. Wiss., Math.-Naturwiss.* **174**, 394–402 (1965).
35. Ganske, A. S., Vahtera, V., Dányi, L., Edgecombe, G. D. & Akkari, N. Phylogeny of Lithobiidae Newport, 1844, with emphasis on the megadiverse genus *Lithobius* Leach, 1814 (Myriapoda, Chilopoda). *Cladistics* **37**, 162–184 (2021).
36. Kovačević, Ž. Prilog fauni Myriapoda Hrvatske. *Glasnik Hrvatskoga Naravnoslovnoga Družtva* **30**, 1–8 (1918).
37. Kovačević, Ž. IV. prilog poznavanju Myriapoda Jugoslavije. *Glasnik Jugoslavenskog Entemološkog Društva* **5**(6), 66–76 (1931).
38. Langhoffer, A. Fauna hrvatskih pećina (spilja) (Fauna cavernarum Croatie) I. *Rad Jugosl. akad. znan. umjet., Mat.-prir. razred* **193**, 339–364 (1912).
39. Langhoffer, A. Fauna hrvatskih pećina (spilja) (Fauna cavernarum Croatie) II. *Prirodoslovna Istraživanja Hrvatske i Slavonije* **7**, 3–22 (1915a).
40. Langhoffer, A. Adatok a horvát barlangi fauna ismeretéhez. *Barlangkutatás* **3**(2), 63–71 (1915b).
41. Pavlova, A. S. Methodical review of the research about cave fauna in western Rhodope, Bulgaria. *Ecol. Balk.* **1**, 103–120 (2009).

42. Polak, S. & Pipan, T. The subterranean fauna of Križna jama, Slovenia. *Diversity* **13**, 210 (2021).
43. Stoev, P. On two myriapods (Chilopoda, Diplopoda) new to the fauna of Albania. *Hist. Nat. Bulg.* **13**, 109–110 (2001b).
44. Stoev, P. *Catalogue and key to the centipedes (Chilopoda) of Bulgaria*. 1–103 (Pensoft, 2002).
45. Vagalinski, B. & Stoev, P. An annotated checklist of the myriapods of the Western Rhodopes (Bulgaria and Greece) in *Biodiversity of Bulgaria 4. Biodiversity of Western Rhodopes (Bulgaria and Greece) II* (ed. Beron, P.) 129– 143 (Pensoft & Nat. Mus. Natur. Hist., 2011).
46. Verhoeff, K.W. Arthropoden aus sudostalpinen Hohlen, gesammelt von Karl Strasser, 2. Aufsatz. *Mitteilungen über Höhlen und Karstfoschung*, 41–55 (1929).
